# Fluidic bacterial diodes rectify magnetotactic cell motility in porous environments

**DOI:** 10.1038/s41467-021-26235-6

**Published:** 2021-10-12

**Authors:** Nicolas Waisbord, Amin Dehkharghani, Jeffrey S. Guasto

**Affiliations:** 1 Department of Mechanical Engineering, Tufts University, 200 College Avenue, Medford, MA 02155 USA; 2 Univ Rennes, CNRS, Geosciences Rennes, UMR 6118, Rennes, F35000 France

**Keywords:** Cellular motility, Biological physics, Fluid dynamics

## Abstract

Directed motility enables swimming microbes to navigate their environment for resources via chemo-, photo-, and magneto-taxis. However, directed motility competes with fluid flow in porous microbial habitats, affecting biofilm formation and disease transmission. Despite this broad importance, a microscopic understanding of how directed motility impacts the transport of microswimmers in flows through constricted pores remains unknown. Through microfluidic experiments, we show that individual magnetotactic bacteria directed upstream through pores display three distinct regimes, whereby cells swim upstream, become trapped within a pore, or are advected downstream. These transport regimes are reminiscent of the electrical conductivity of a diode and are accurately predicted by a comprehensive Langevin model. The diode-like behavior persists at the pore scale in geometries of higher dimension, where disorder impacts conductivity at the sample scale by extending the trapping regime over a broader range of flow speeds. This work has implications for our understanding of the survival strategies of magnetotactic bacteria in sediments and for developing their use in drug delivery applications in vascular networks.

## Abstract (web summary)

Microswimmers can navigate porous environments, however the impact of their directed motility on their movement in fluid flow remains an open issue. The authors show that the motility of magnetotactic bacteria in flow through a porous constriction gives rise to nonlinear flow conductivity similar to electrical diodes.

## Introduction

Hydraulic networks, ranging from saturated soils and sediments to human tissues and filtration media, play host to diverse swimming microbes^1–3^. To navigate these complex geometries, cells use environmental cues to direct their motility towards favorable conditions via an array of sensing (e.g., chemotaxis)^4–6^ and physical reorientation mechanisms (e.g., magnetotaxis)^7^. However, directed cell motility can be modified by ambient fluid flows^8–11^, which profoundly affects biomass distribution and productivity in the environment^6,8^. The geometric confinement of these environments regulates fluid flow and consequently, cell growth^12^ and swimming dynamics^13–15^. Confinement thus plays a central role in coupling directed motility to flow^16–19^. For example, in porous media flows, chemotaxis significantly augments the retention of swimming bacteria^13,20^. Directed microbial motility in porous geometries is integral to ecological, environmental, and biomedical applications^3,21^. Yet the impact of the microscopic coupling between directed cell swimming and heterogeneous flows on sample-scale cell transport properties remains elusive^22^.

Magnetotactic bacteria (MTB) naturally biomineralize nano-scale magnets called magnetosomes, which are embedded in their cell membrane. These particles are arranged in a linear array and form a permanent magnet that mechanically and passively aligns MTBs with Earth’s magnetic field^7^, enabling them to seek out microaerobic conditions in heterogeneous marine sediments^23^. MTBs provide an ideal physical^18^ and relevant ecological^23,24^ model system to probe the effects of directed motility on cell transport in heterogeneous flows: Their primary directional cue—the magnetic field—is purely mechanical, uniform, and independent of the ambient flow field. Beyond gaining insight into the evolutionary advantages of natural magnetotactism, MTBs are a promising candidate in biomedical applications for targeted drug delivery due to their potential to be directed through heterogeneous vascular network flows^25,26^.

**Fig. 1:**
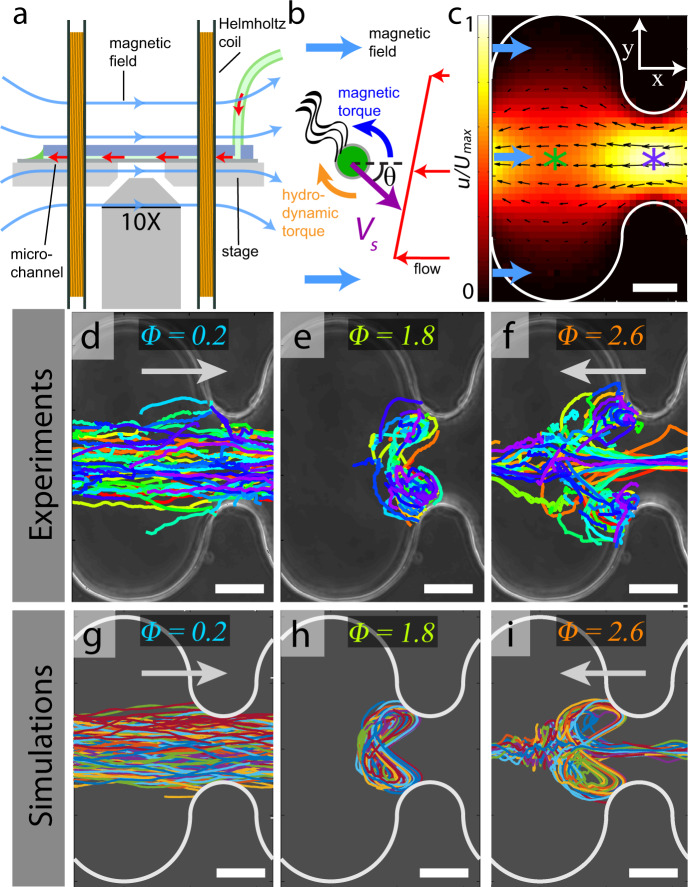
Porous media flows trap upstream swimming magnetotactic bacteria in vortical trajectories. **a** Schematic of the experimental set up. Helmoltz coils generate a uniform magnetic field (blue arrows) to direct magnetotactic bacteria (MC-1) upstream against a pressure-driven microfluidic flow (red arrows). **b** Schematic of the torques acting on the magnetotactic bacteria. Hydrodynamic torque (orange) due to the local velocity gradient (red) opposes a magnetic torque (blue) due to misalignment of the cell swimming orientation (purple) relative to the magnetic field (light blue). **c** Measured flow field inside of a corrugated microfluidic channel (*w*_*p*_/*w*_*t*_ = 5; Supplementary Fig. 2). Colormap indicates normalized flow speed and markers indicate the location of maximum (*U*_max_; purple) and minimum (*U*_min_; green) centerline flow speed. **d**−**f** Experimentally measured cell trajectories (147, 103, and 145 tracks) in a corrugated channel (*w*_*p*_/*w*_*t*_ = 5; see also Supplementary Movies 1−3) corresponding to maximal flow speeds *U*_max_ = 30, 260, and 350 μm/s. Gray arrows indicate the direction of bacterial transport. Scale bar, 40 μm. **g**−**i** Langevin simulations (50 tracks each) corresponding to conditions in (**d**−**f**), respectively. Scale bar, 40 μm.

In this study, we use microfluidic experiments (Fig. 1) in 1D porous media to unveil a novel, pore-scale transport mechanism for dilute suspensions of magnetotactic bacteria: cells, directed to swim upstream against a continuous, externally controlled flow, become trapped in striking vortical orbits when traversing a constriction. Vortical localization leads to highly nonlinear bacterial flux through a pore, characterized by three distinct flow regimes: similar to an electrical diode^27^, the transport is completely halted for a wide range of flow rates. This bacterial diode phenomenon is in stark contrast to linear fluidic network and continuum transport models^28,29^, which typically stem from a detailed understanding of pore-scale processes^30–32^. Simulations, based on single-cell motility modeling, reveal that cell trapping due to vortical localization is associated with diverging residency times and enhanced dispersion. We experimentally show that vortical trapping persists in higher dimensions (3D packed beds). Further, we extend our simulations to illustrate how disorder greatly broadens the trapping regime to impact sample scale transport, due to the dominance of a few strongly trapping pores. These nonlinear transport properties pose new challenges for modeling directed active matter^15,33^, far beyond the paradigms of classical passive scalar transport^34^.

## Results

### Vortical cell trapping at constrictions

A suspension of north-seeking *Magnetococcus marinus* (MC-1) bacteria^35^ (swimming speed, *V*_*s*_ = 135 ± 25 μm/s; Supplementary Information) is magnetically guided upstream against a continuous, externally controlled flow through successive pores of a corrugated microchannel, unveiling highly nonlinear directed transport. The dilute concentration of bacteria ( ~10^6^ cells/ml; see “Methods”) ensures that cell−cell interactions are negligible^36^. The channels (height, *h* = 110 μm)^37,38^ comprise semi-circular walls with alternating concavity (Fig. 1c; Supplementary Fig. 1) to form a series of constrictions (throats) and expansions (pores). The throat walls have a fixed radius of curvature (35 μm) and separation distance, *w*_*t*_ = 70 μm, while the radius of curvature of the pore walls is adjusted to vary the pore width, *w*_*p*_ = 210, 280, and 350 μm. The flow field in the micro-channel is measured by particle tracking velocimetry (Fig. 1c and Supplementary Fig. 2; see also “Methods”), and the flow strength is characterized by the maximum, *U*_max_, and minimum, *U*_min_, centerline flow speeds, measured in the throat and pore, respectively. The magnetic field direction is imposed by a set of Helmholtz coils adapted to the microscope (Fig. 1a), which generate a strong magnetic field strength, *B* = 1.75 mT ( ≈30 times Earth Magnetic Field). The restoring magnetic torque on the cells competes with stochastic and hydrodynamic body reorientations and aligns the MTB swimming direction upstream.

Starkly different cell trajectories are observed for various flow regimes, parameterized by the maximal channel flow speed normalized by the mean cell swimming speed, Φ = *U*_max_/*V*_*s*_ (Fig. 1d−f). Hydrodynamic torque from the local vorticity modifies the cell orientation and thus swimming dynamics^8,9,39^ (Fig. 1b). For small flow speeds, the magnetic torque maintains bacterial orientation and enables upstream swimming against the flow (Fig. 1d, Φ = 0.2; Supplementary Movie 1). For large flow speeds, cells are consequently swept downstream (Fig. 1f, Φ = 2.6; Supplementary Movie 3). However, for intermediate flow speeds, the bacteria form localized, vortical swimming orbits (Fig. 1e, Φ = 1.8; Supplementary Movie 2) that persists across pore geometries (*w*_*p*_/*w*_*t*_ = 3, 5, 7) and are evident in the spatial distribution of cells (Supplementary Fig. 4). By accounting for hydrodynamic and magnetic torques on individual cells (Fig. 1b), we developed a Langevin model of magnetotactic motility^18^ (see “Methods” and Supplementary Information; Supplementary Fig. 3), which accurately predicts our experimental observations (Fig. 1g–i; Supplementary Fig. 4). The Langevin model also enables us to explore a broader range of ecologically relevant magnetic field strength (Supplementary Fig. 6) and alignment conditions (Supplementary Fig. 7) and to quantify the long-time cell transport properties of this system, relative to what is achievable by experiments alone.

### Pores act as fluidic diodes for upstream swimming cells

**Fig. 2:**
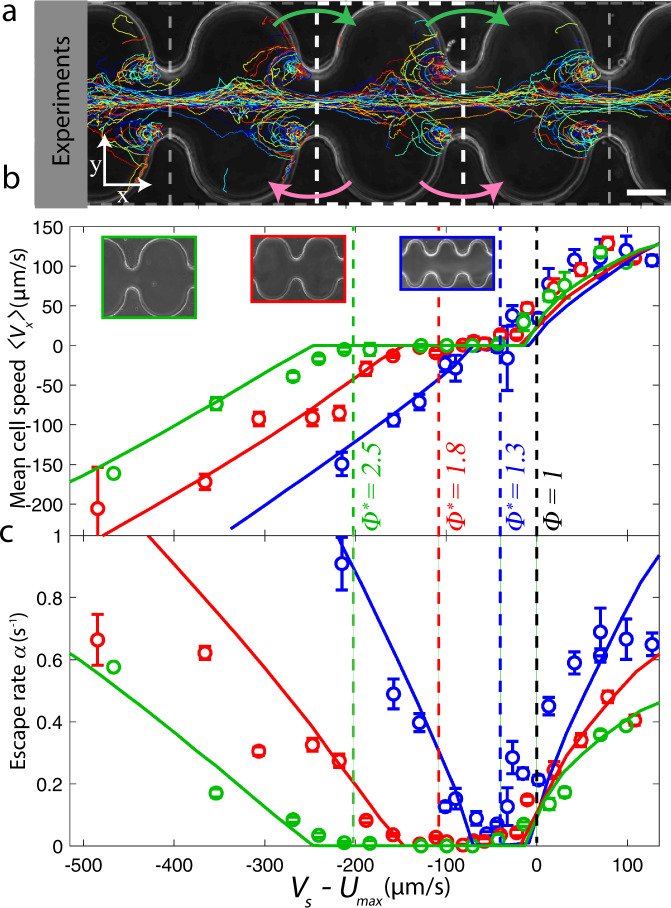
Cell trapping at a constriction results in a bacterial diode effect, having three distinct transport regimes. **a** Corrugated microchannels (*w*_*p*_/*w*_*t*_ = 5; Φ = 2.8) enable periodic averaging of cell transport statistics from experimentally measured magnetotactic bacterial trajectories (670 tracks shown). Gray dashed lines represent periodic pore edges. Cell flux through a pore control volume (white dashed lines) in the upstream direction (green arrows) and positive cell flux out of a pore, *J*_out_ (magenta arrows) determine the mean cell advection speed, 〈*V*_*x*_〉, and pore escape rate, *α*, respectively. Scale bar, 50 μm. **b** Mean upstream cell advection speed, 〈*V*_*x*_〉, exhibits three transport regimes: upstream swimming, trapping, and downstream advection. Markers are experiments (*w*_*p*_/*w*_*t*_ = 3, blue; *w*_*p*_/*w*_*t*_ = 4, red; *w*_*p*_/*w*_*t*_ = 5, green), and solid lines are corresponding Langevin simulations. Vertical dashed black line corresponds to predicted limit of the upstream regime, Φ = 1 (*V*_*s*_ = *U*_max_). Vertical dashed colored lines are predicted limits of the trapping regime, Φ^*^ = *U*_max_/*U*_min_ (*V*_*s*_ = *U*_min_). **c** Bacterial pore escape rates (see **a**), *α*, reflect the non-marginal cell trapping. Markers and lines correspond to (**b**). Error bars for (**b** and **c**) are standard errors of 9, 5, and 4 individual pores along the corrugated channel acquired simultaneously for *w*_*p*_/*w*_*t*_ = 3, 4, and 5, respectively. Each data point is comprised of 3667 tracks on average with a minimum of 254 tracks across all data.

Linearity of fluid and solute transport with the driving pressure is a hallmark of most conventional hydraulic networks^40^. While bacterial transport is often modeled by simple advection-diffusion processes^4^, it is not clear whether linear conductivity is maintained in the case of directed swimming cells^16–18^ (Fig. 1d−i). The mean bacterial advection speed, 〈*V*_*x*_〉, through a pore (Fig. 2a) reveals three distinct transport regimes across different continuous flow strengths, *U*_max_, which bears a striking resemblance to the forward, backward, and breakdown regimes for current flow through an electrical diode^27^. For a fixed magnetic field and pore geometry, we find: (i) In the upstream regime (Φ = *U*_max_/*V*_*s*_ < 1), the swimming speed is large compared to the flow speed (Fig. 2b, black dashed line), and MTB swim upstream with a net positive mean advection speed, 〈*V*_*x*_〉 > 0. (ii) The trapping regime (1 < Φ < Φ^*^) is characterized by a near-zero mean bacterial advection speed, 〈*V*_*x*_〉 ≈ 0, and coincides with the emergence of bacterial vortices (Fig. 1 e, h). As cells approach the throat, a hydrodynamic torque orients and concentrates the cells toward the channel center line^18^. Because the throat speed surpasses the swimming speed, *V*_*s*_ < *U*_max_, cells are gated from passing the throat and are swept downstream. Once in the low-speed pore region where *V*_*s*_ > *U*_min_, cells escape from the centerline and swim back upstream against the flow, repeating the cyclic orbit. Thus, *V*_*s*_ = *U*_min_ sets the critical non-dimensional flow speed Φ^*^ = *U*_max_/*U*_min_, which defines the trapping regime (Fig. 2b, colored dashed lines) and increases with pore-throat aspect ratio, *w*_*p*_/*w*_*t*_. (iii) In the downstream regime (Φ > Φ^*^), the flow speed dominates cell swimming speed even in the pore region, *V*_*s*_ < *U*_min_. Bacteria are swept downstream with a negative mean bacterial advection speed, 〈*V*_*x*_〉 < 0, and the vortical orbits begin to dissipate. In contrast to the linear hydrodynamic conductivity of the carrier fluid (Supplementary Fig. 2), the flow conductivity of directed bacteria through confined pores is highly nonlinear and completely stifled in some cases.

Langevin simulations show that the diode-like transport behavior persists across a broad range of magnetic field strength (Supplementary Fig. 6) and alignment (Supplementary Fig. 7) conditions. Specifically, the strength of the magnetic field dictates the size of the trapping vortices and the efficacy of the trapping. The relatively high magnetic fields investigated in experiments dominate over the rotational noise of the cells, but as the magnetic field strength decreases, cells take larger lateral excursions in the pores (Supplementary Fig. 6f−t). This results in a dilation of the trapping vortices and a reduction in the range of the trapping regime (Supplementary Fig. 6a−e). Physically, as the trapping vortex size becomes larger than the pore size, the trapping strength diminishes. Notably, the diode phenomenon persists at Earth’s magnetic field strength (Supplementary Fig. 6e). In general, the flow direction is not necessarily aligned with the magnetic field direction. Perturbations from the antiparallel orientation explored in experiments reveal that as the angle between the magnetic field and flow directions is increased, one trapping vortex is diminished in favor of the other (Supplementary Fig. 7k−o), but the bacterial diode conductivity profile is preserved (Supplementary Fig. 7a−e). These results suggest that the nonlinear conductivity of directed microbes is robust and relevant to both the physical ecology of MTB in the natural environment^7,23,24^, and potential biomedical applications^26^.

Despite the stochastic nature of bacterial motility^4^, the observed vortical localization of the cells suppresses not only their mean advection speed but also the cell escape rate from a pore (Fig. 2c). The statistics of the escape rate, *α*, reflect the three observed flow regimes for the bacterial conductivity (Fig. 2b), where *α* = *J*_out_/*N*_in_, *J*_out_ is the total cell flux out of a pore, and *N*_in_ is the number of cells in the pore (Fig. 2a). For weak flows (Φ < 1) and for strong flows (Φ > Φ^*^), the escape rate is large owing to the dominance of upstream swimming and downstream advection, respectively. For intermediate flow speeds (1 < Φ < Φ^*^) in the trapping regime, the escape rate decreases to nearly zero, indicating suppressed transport. Beyond the qualitative agreement in the swimming trajectories (Fig. 1d−i), the strong quantitative agreement between simulations and experiments in capturing 〈*V*_*x*_〉 and *α* demonstrates the predictive value of our Langevin model (Fig. 2b, c), which we use to study the long-time dispersive properties of these directed active suspensions.

### Pore-scale trapping increases retention time

**Fig. 3:**
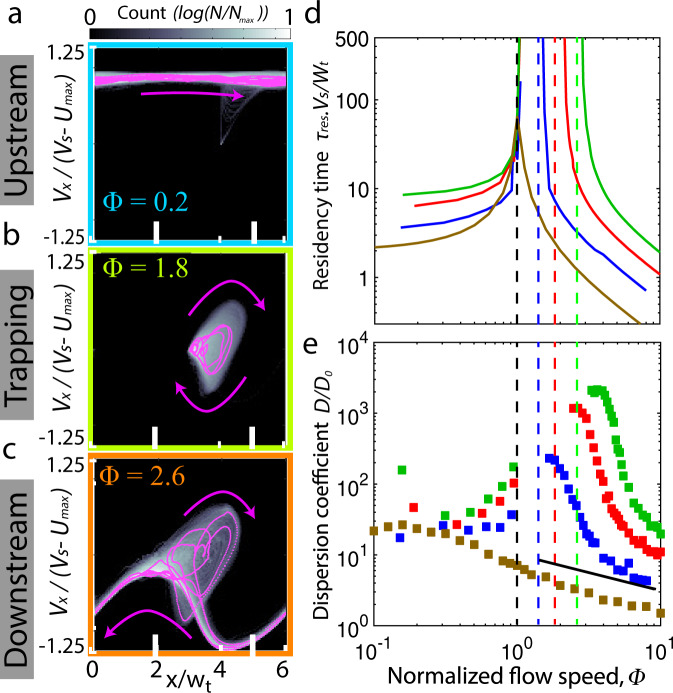
Vortical trapping enhances retention and increases dispersion. **a**−**c** Phase density of cell trajectories from simulations (*w*_*p*_/*w*_*t*_ = 5; see also Supplementary Fig. 5) for three observed transport regimes: upstream (**a**, Φ = 0.2), trapping (**b**, Φ = 1.8), and downstream (**c**, Φ = 2.6). A magenta curve is a single, sample cell trajectory with arrows indicating direction. Color map shows the normalized cell count (log-scale) for 100 cells over a simulated time *t* = 500*w*_*t*_/*V*_*s*_. **d** Normalized residency time in a pore as a function of normalized flow speed Φ determined from Langevin simulations for *w*_*p*_/*w*_*t*_ = 3 (blue), 5 (red), and 7 (green). Brown line corresponds to a straight duct (width, *w* = *w*_*t*_). Vertical black and colored dashed lines correspond to Φ = 1 and Φ^*^ in Fig. 2b, c. **e** Normalized stream-wise dispersion coefficients, *D*/*D*_0_, as a function of normalized flow speed determined from Langevin simulations for *w*_*p*_/*w*_*t*_ = 3 (blue), 5 (red), and 7 (green) and through a straight duct (brown). All dispersion coefficients are normalized by *D*_0_ for a straight, quiescent duct (Φ = 0). The solid black line indicates slope −1/2.

Retention of bacteria in porous media has been extensively studied for its environmental and ecological importance^41^. The dynamical cell trapping investigated here leads to a new mechanism for MTB retention in porous media flows. Phase-space analysis of simulated cell trajectories reveals the swimming dynamics responsible for the modified transport of directed MTB (Fig. 3a−c; see also Supplementary Fig. 5). In the upstream regime, cells migrate unimpeded, and their upstream speed fluctuates within a positive range (*V*_*x*_/(*V*_*s*_ − *U*_max_) > 0) as they pass through low-speed pores and high-speed throats (Fig. 3a) with normalized cell position, *x*/*w*_*t*_. As the flow speed increases and the system reaches the trapping regime, vortical cell trajectories (Fig. 1e, h) emerge as bounded orbits in this phase space, with their speed alternating sign as they traverse irregular loops (Fig. 3b; Supplementary Fig. 5). The residency time within a pore thus diverges, corroborating the *α* ≈ 0 experimentally measured escape rates (Fig. 2c). In the downstream regime, cells begin to escape the vortical orbits (Fig. 3c, upper lobe; Supplementary Fig. 5). Subsequently, the cells are advected downstream, where they sample strictly negative advection speeds (*V*_*x*_/(*V*_*s*_ − *U*_max_) < 0) on the lower branch of the distribution. Individual cells switch between these two states in a stochastic catch-and-release process.

For the directed MTB motility in porous media investigated here, the average residency time, *τ*_res_ = 1/*α*, in a pore increases as the normalized flow speed approaches Φ = 1 for every geometry, corresponding to 〈*V*_*s*_〉 = 0 (Fig. 3d). For comparison, we also examine the MTB residency time in a straight duct, where the effective pore size is defined as a square region with a side length corresponding to the duct width. In the straight duct, the cells are focused in a narrow beam centered in the channel^18^, and the normalized residency time peaks at a finite value, *τ*_res_*V*_*s*_/*w*_*t*_ = 30, for Φ = 1. Despite the average cell speed going to zero around Φ = 1, their continuous swimming and random reorientations cause the cells to eventually escape the boundaries defined above. For the corrugated channels, on the other hand, there exists a finite regime of 1 ≤ Φ ≤ Φ^*^ over which the normalized retention time appears to diverge and no cells escape within the simulation time, *τ* ≥ 20,000.

These results identify a new mode of bacterial retention at the pore scale, due to the dynamical trapping of swimming magnetotactic cells. Recently, it has been shown that bacterial retention in porous media flows is enhanced by chemotaxis toward contaminants in the medium^20^, and likewise, retention is reduced when rinsing with a chemoattractant^13^. Such chemotactic retention mechanisms are inherently transient, due to both the driving chemoattractant concentration field and bacterial distribution being coupled to the ambient flow. In contrast, the dynamical trapping of MTB shown here is omnipresent, owing to the independence of the directing magnetic field. We expect that this fundamentally new retention mechanism will work in concert and competition with a chemotaxis^13,20^ and a range of other-directed swimming motilities in confined flows. Retention and intermittent trapping are also expected to enhance the dispersion of the cell population, beyond their random walk motility.

### Anomalous dispersion in corrugated channels

Langevin simulations demonstrate that the MTBs dispersion can be drastically enhanced or suppressed, depending strongly upon the details of the pore-scale flow. Bacterial dispersion coefficients, *D*, were measured from the linear regime of the mean square displacements along the flow direction, *MSD* = 〈(Δ*x* − 〈Δ*x*〉)^2^〉 and normalized by the effective diffusion coefficient, *D*_0_, without flow in a straight duct (Fig. 3e). (Fig. 3d, black lines). For comparison with the corrugated channels, we also examine the dispersion of MTB in a straight duct (Fig. 3e, brown), where the normalized dispersion scales as *D*/*D*_0_ ~ Φ^−1/2^ due to a well-established flow-focusing effect along the flow centerline^16–18^. Corrugation is well known to enhance the dispersion of Brownian particles^38^, and in the upstream regime, the dispersion coefficient of the actively swimming MTB initially grows with increasing flow speed. However, once the trapping regime (1 < Φ < Φ^*^) is reached, *D*/*D*_0_ can no longer be defined in our simulations, since the trapping time is longer than the simulation time (Fig. 3d), and the diffusive regime is never reached. In contrast, this is not the case for the straight duct, which always exhibits a finite residency time. As the residency times become finite again in the downstream regime (Φ > Φ^*^), the diffusive behavior is recovered and exhibits a strikingly large dispersion coefficient (up to *D*/*D*_0_ ≈ 4 × 10^3^; Fig. 3e). The observed dispersion enhancement stems from the catch-and-release of cells through ephemeral retention in localized vortices (Fig. 3c), which drastically spreads out the bacterial population. For Φ ≫ 1, *D*/*D*_0_ asymptotically decays to the dispersion in a straight duct. The non-monotonic behavior of the dispersion coefficient stands in stark contrast to classical Taylor dispersion^34^, further illustrating the challenges for analytical models of active matter transport^15,19^.

### Vortical trapping in disordered geometries of higher dimensions

**Fig. 4:**
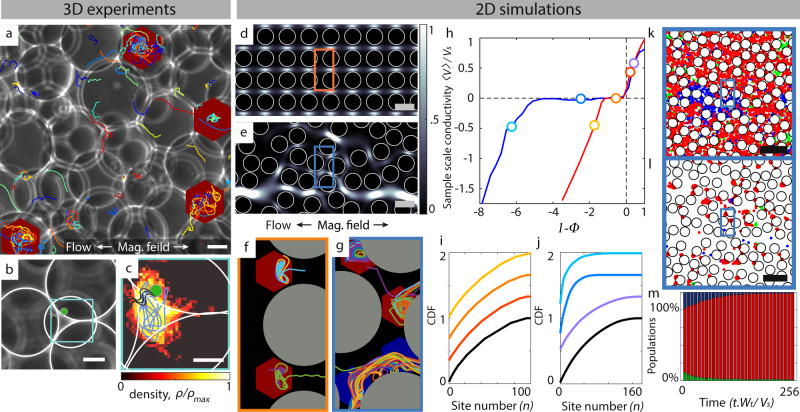
Vortical trapping in disordered geometries of higher dimension. **a** Magnetotactic bacteria trajectories strongly localize in specific pores (red hexagons) of a microfluidic packed bed of hydrogel beads. White arrows indicate magnetic field (1.75 mT) and mean flow directions. Scale bar, 10 μm. **b** Sample pore containing a single, trapped cell (indicated by cell sketch). The tetrahedral pore is comprised of a triad of in-plane beads and one out of plane (center). Scale bar, 10 μm. **c** Distribution of positions for a single localized cell, tracked for 6.5 min (cyan square from **b**; Supplementary Movie 5). The blue curve is a 20 s long sample segment of the trajectory, and the colormap indicates the cell’s distribution over time. Scale bar, 5 μm. **d**, **e**, 2D flow fields through regular and disordered arrays of cylindrical pillars. **f**, **g** Cell trajectories in ordered and disordered media. Flow conditions correspond to colored boxes in (**f** and **g**) and red and blue markers in the sample scale trapping regime of (**h**), respectively. Red and blue hexagons mark local pores in the trapping and upstream regimes, respectively. **h** Sample scale conductivity 〈*V*_*x*_〉/*V*_*s*_ for ordered (red curve) and disordered (blue curve) geometries, as a function of the flow strength for steady-state transport conditions. For each curve, regimes with 〈*V*_*x*_〉/*V*_*s*_ ≈ [0.5, 0, −0.5] are marked by circles. **i**, **j**, Cumulative distribution functions (CDFs) of number of cells per trapping site (pore) in the ordered and disordered geometries, for Φ = 0 and 〈*V*_*x*_〉/*V*_*s*_ ≈ [0.5, 0, −0.5]. Colors refer to specific regimes marked in (**h**). CDFs are artificially offset for clarity. **k**, **l** Location of cells for 〈*V*_*x*_〉/*V*_*s*_ = 0 in the disordered geometry for initial and steady-state conditions. Colored dots mark cells in the upstream (green), trapping (red), and downstream (blue) regimes. **m** Populations of cells in the upstream (green), trapping (red), and downstream (blue) regimes as a function of time for the disordered geometry for 〈*V*_*x*_〉/*V*_*s*_ = 0.

Natural microbial habitats are characterized by flows through heterogeneous pore structures^31,42^, far more complex than the periodic, one-dimensional geometry in which vortical trapping of MTBs was identified above (Figs. 1 and 2). Here, we experimentally demonstrate that this pore-scale effect occurs in disordered and higher dimensional porous media. Polydisperse, transparent hydrogel beads (10−25 μm radius) are randomly packed under flow into a rectangular microfluidic channel (height, 75 μm; width, 1000 μm; Supplementary Information). In a quiescent environment, swimming MTBs invade the entire medium. However, imposing an ambient flow and an opposing magnetic field reveals that only specific pores emerge as trapping sites (Fig. 4a, red hexagons; Supplementary Movie 4), where cells remained for up to several minutes (Fig. 4c; Supplementary Movie 5). In these trapping sites, cells appear to swim along more erratic trajectories compared to the regular vortexes observed in the 1D medium (Fig. 4c), likely due to the 3D flow geometry. Despite the more complex dynamics, the distribution of cell positions within the trapping pores remains narrow (Fig. 4c) at about half of the bead diameter, ≈20 μm. While we cannot entirely rule out other effects, the observed localization appears consistent with the hydrodynamic trapping demonstrated in the 1D corrugated microfluidic channels. Importantly, cells only localize in sporadic pores throughout the medium, suggesting that the heterogeneity of the flow field translates into the coexistence of different diode transport regimes (Fig. 2).

To elucidate the impact of microstructural heterogeneity on MTB transport, we adapt our simulations (see “Methods”) established for the corrugated channels to two different 2D geometries: (i) a regular square lattice of cylindrical obstacles (Fig. 4d) and (ii) a disordered version (Fig. 4e). Both geometries are comprised of 100 obstacles and have a porosity of 0.5. As in the 1D and 3D experiments, vortical trapping is readily observed over a finite range of sample-scale mean flow speeds through the 2D structures (Fig. 4f, g). The positions of these trapping pores are identified in the geometry as regions of high residency time for at least one of the sample-scale mean flow speeds. For the ordered lattice, they appear on the downstream side of the throats between obstacles, where vortexes form when the local pore reaches its trapping regime (Fig. 4f). Due to the high degree of lateral symmetry, the MTB form dual vortexes that exhibit a figure-8 shape, similar to the 1D corrugated channels (Fig. 1). These trapping regions are detected as a single high residency time site in our analysis, giving 100 detected traps for 100 obstacles. In this geometry, all of the pores are identical, and for a given mean sample scale flow speed, they simultaneously exist in one of the three observed diode regimes identified for the 1D corrugated channels. Thus, the nonlinear diode-like conductivity curve persists for directed MTB transport in the ordered 2D lattice (Fig. 4h, red curve).

In the disordered geometry, the same three distinct transport regimes are also observed, but the sample scale conductivity exhibits a marked five-fold increase in the range of the trapping regime (1≤Φ ≲ 6; Fig. 4h, blue curve). At the pore scale, adjacent pores simultaneously exist in different transport regimes (see Fig. 4g) with different pores entering and exiting the trapping regime as the sample scale mean flow speed increases (Supplementary Fig. 8). The broken symmetry of the throat flow direction with the magnetic field direction biases the trapping regions toward one side of the throat, which is consistent with 1D simulations in the corrugated channel for non-parallel magnetic field directions relative to the flow (Supplementary Fig. 7). Thus, the pore-scale heterogeneity extends the range of the sample scale trapping regime (Fig. 4h, blue curve). Cell seeding  is homogeneous in the porous media. Due to the periodic boundary conditions, cells circulate through the structures passing through pores in the upstream and downstream regimes, until they encounter a pore in the trapping regime (Fig. 4k, l). Over time, the cell concentration in the strongest trapping pores increases (Fig. 4m). As a result, cells densify in only a fraction of the available high residency sites for the sample scale trapping regime (Fig. 4i, j), while no such selection is observed for the ordered geometry. In disordered media, the local flow conditions that dictate the efficacy of dynamical trapping for directed swimming cells are not only a function of the sample-scale flow but also the local pore-throat structure.

## Discussion

Linear hydraulic network theory has been instrumental to our understanding of fluid and solute transport in porous media^28,40^, but active matter poses a host of new theoretical challenges^12,15,19,39^ when compared to Brownian particle transport^43,44^. Our results illustrate how the directed motility of individual swimming microorganisms couples to pore geometry and flow, resulting in highly nonlinear dispersion and flow conductivity. The retentive properties of these directed bacterial flows draw parallels to the conductivity of electric diodes, as opposed to the usual analogy of resistor networks for a simple fluid flowing through porous media. While future experiments are necessary to fully discern the effects of magnetic field strength and porous microstructure, the pore-scale trapping of directed, swimming microbes observed here carries clear implications for microbial ecology and evolution. For magnetotactic bacteria, localization in porous sediments^23,24^ may facilitate niche exploitation through increased residence time and thus the uptake of dissolved matter^6^. Moreover, these results inform potential control strategies for genetically engineered magnetotactic^25,26^ and synthetic microswimmer guidance^45^ in medical applications. We expect that our results are broadly applicable to other physical mechanisms of directed motility that rely on an aligning torque, including gyrotaxis^8^ for which the directed motility in heterogeneous turbulent flows also leads to the accumulation of organisms^46^. Sensory-based cell navigation is additionally complicated, for example in chemotaxis, by the coupling of the chemical landscape to fluid advection^9,11,13,20^. However, phototactic phytoplankton exhibit similar flow coupling behavior^17^ to magneto-^18,33^ and gyro-tactic^16^ microbes, suggesting that this observed nonlinear microbial transport mechanism is indeed widespread. Although the diode phenomenon illustrated here arises from single-cell motility, cell−cell interactions due to a local or global increase in cell concentration are expected to further enrich the nature of microbial transport under confinement^47,48^. Elucidating the impact of porous media flows on collective cell transport represents an exciting new avenue for this work.

## Methods

### Cell culturing

Magnetotactic bacteria (*Magnetococcus marinus*, MC-1) are cultivated in standard agarose gels within a chemically imposed oxygen gradient, using well-established protocols^35^. A stock solution of artificial sea water (16.4 g/l of NaCl, 3.5 g/l of MgCl_2_, 2.8 g/l of Na_2_SO_4_, 0.5 g/l of KCl, 1 g/l of CaCl_2_) is prepared in a 1 l glass bottle. 100 ml is sampled from this stock solution, to which the following are added: 0.2 g of HEPES, 0.03 g of NH_4_Cl, 0.5 μl of Wolfe mineral solution, 80 μl of 0.5% resazurin solution, and 300 μl of 0.01 M FeS0_4_ solution. The solution is completed with 180 μl of 0.5 M phosphate buffer (pH 6.8) and 0.16 g of agarose. Separately, 1 g of sodium bicarbonate is inserted into a tightly screwed glass tube. Both the 100 ml solution and the sodium bicarbonate tube are autoclaved. The solution is cooled to 60 ^∘^C, and 560 μl of a 20% sodium thiosulfate solution, 40 mg of fresh cysteine, and 280 μl of a 10% sodium bicarbonate solution are added. Before gelation, the solution is poured into a 16 ml glass test tube, leaving 1 cm of air space, and capped. An oxygen gradient forms via diffusion and is visualized by the presence of resazurin. Cells grow at the oxic−anoxic transition zone (OATZ), where they form a heterogeneous growth front. After three days of growth, ≈1 μl of cells is sampled from the front via pipette and deposited at the microfluidic channel inlet. Cells are then directed into the corrugated microchannel via an applied magnetic field. These conditions result in a dilute cell concentration of ~10^6^ cells/ml, which enables the tracking of individual cells. Motility media, which is a liquid version of the growth media (without agarose, sodium bicarbonate, and cysteine), was used for flow experiments.

### Microfluidics

Microfluidic channels were fabricated from polydimethylsiloxane (PDMS) using standard soft lithography techniques and plasma bonded to standard 1 mm glass slides. The flow speed in the channels (0 ≤ *U*_max_ ≤300 μm/s ± 2 μm/s) is regulated using a precision pressure controller (Elveflow OB-1), where the dynamic range (0 ≤ *P* ≤ 150 mbar) is set by an additional resistor channel (Supplementary Fig. 1) and calibrated for each channel geometry (Supplementary Fig. 2). Microfluidic packed beds were formed by jamming a suspension of crosslinked agarose hydrogel beads (Agarose Bead Technologies) in a straight microfluidic channel having a constriction at one end (0.4−0.44, estimated porosity). For each experiment, the motility media is injected at one end of the channel, and the cells are magnetically guided upstream through the channel from the open outlet.

### Video microscopy and cell tracking

Cells were imaged using phase-contrast microscopy (10×, 0.3 NA; Nikon Ti-e), and videos of cell motility (≥400 frames each) were captured (Andor Zyla camera) at frame rates ≥30 frames/s. Cells were tracked using a predictive particle tracking algorithm implemented in a custom MATLAB code^9^. Cell trajectories lasting more than ≥0.3 s were analyzed, and cell speeds were obtained using a Savitsky−Golay filter over five video frames.

### Flow field measurement

Flow fields (Fig. 1c) in the corrugated channels are measured by particle tracking velocimetry (PTV). Following cell motility experiments, cells were washed from the channel, and a dilute suspension of fluorescent tracer particles (0.5 μm diameter; Invitrogen) was introduced into the channel. Tracers were flowed at a range of applied pressures (Supplementary Fig. 2) and imaged using fluorescence microscopy (10×, 0.30 NA objective). Measured particle velocities (2000 frames per applied pressure condition) were binned in a regular grid to obtain the velocity field.

### Langevin simulations

A Langevin model of swimming MTB^9,18,39^ was constructed by considering the forces and torques (Fig. 1b) acting upon on a single cell resulting in translational and rotational dynamics. For each geometry (1D corrugated channels and 2D porous structures) and flow condition, individual bacterial trajectories were computed numerically by solving the stochastic equations of motion in the plane:

1 $$\dot{x}={V}_{s}\cos \theta +u(x,y)$$

2 $$\dot{y}={V}_{s}\sin \theta +v(x,y)$$

3 $$\dot{\theta }=\frac{\omega (x,y)}{2}+{\mu }_{r}MB\sin (\theta )+\sqrt{2{D}_{r}}\xi (t)$$

The translational dynamics of the cells are modeled by a superposition of swimming speed, *V*_*s*_, and local fluid velocity, *u* and *v*. The orientational dynamics are governed by hydrodynamic rotation due to vorticity, *ω*, magnetic field alignment due to a magnetic torque, and rotational noise that encompasses Brownian rotational diffusion and flagellar-induced orientational fluctuations of the cells (Fig. 1b). The flow field, *u* and *v*, and vorticity, *ω*, are determined from finite element simulations in each geometry (Supplementary Information and Supplementary Fig. 2) and vetted by particle tracking velocimetry measurements (Fig. 1c). The ensemble average effective rotational diffusion coefficient of the cells, *D*_*r*_ = 0.25 ± 0.05 s^−1^, is measured from MTB swimming trajectories in the absence of both external flow and applied magnetic field (Supplementary Fig. 3). While the magnetic moment, *M*, and the hydrodynamic rotational mobility of the cells, *μ*_*r*_, are not readily accessible individually, their product is easily obtained from cell tracking measurements in a calibrated magnetic field (Supplementary Fig. 3). The equations of motion are integrated by a fourth-order Runge-Kutta method. In the corrugated channels, the total dimensionless simulation time was set to *τ* = *tV*_*s*_/*w*_*t*_ = 20,000 with a time step *δτ* = 0.005. In the 2D structures, the total dimensionless simulation time was set to *τ* = *tV*_*s*_/2*R* = 256, 5 times the necessary time for a cell to swim from one end to the other. Steric boundary conditions prevent cells from entering the solid surfaces in our simulations. A Weeks−Chandler−Andersen repulsive potential was implemented:

4 $${{{{{{{{\bf{F}}}}}}}}}_{W}({{{{{{{\bf{r}}}}}}}})=-\nabla {U}_{r}({{{{{{{\bf{r}}}}}}}})$$

5 $${U}_{r}(d)=\left\{\begin{array}{ll}4\epsilon \left[{\left(\frac{a}{d}\right)}^{1/6}-{\left(\frac{a}{d}\right)}^{1/12}\right]&+\epsilon ,d < {2}^{1/16}a\\ &0,d\ge {2}^{1/16}a\end{array}\right.$$

where *d* is the distance to the closest solid surface, *a* is the bacterial radius, and *ϵ* is the hardness (see Supplementary Information for details).

### Reporting summary

Further information on research design is available in the Nature Research Reporting Summary linked to this article.

## Supplementary information

Supplementary Information

Supplementary Movie 1

Supplementary Movie 2

Supplementary Movie 3

Supplementary Movie 4

Supplementary Movie 5

Reporting Summary

## Data Availability

The figure source data files generated in this study have been deposited in the Harvard Dataverse database 10.7910/DVN/YR7CPE. The raw experimental data (cell trajectories in time) and simulation data are available from the corresponding authors upon reasonable request.

## Code availability

The codes associated with source data generated in this study have been deposited in the Harvard Dataverse database 10.7910/DVN/YR7CPE. All other codes used in this study are available from the corresponding authors upon reasonable request.
