# Supplementary material for: Fluidic bacterial diodes rectify magnetotactic cell motility in porous environments
Source: Nat Commun. 2021 Oct 12;12:5949. doi: 10.1038/s41467-021-26235-6 (PMC8511139; doi:10.1038/s41467-021-26235-6)
Supplement: Supplementary file 1 — Supplementary Information [file 41467_2021_26235_MOESM1_ESM.pdf]

Supplementary Information: Fluidic bacterial diodes rectify magnetotactic cell motility in porous environments

Nicolas Waisbord[1,2,*], Amin Dehkharghani[1], and Jeffrey S. Guasto[1,*]

[1]Department of Mechanical Engineering, Tufts University,
200 College Avenue, Medford, MA 02155, USA

[2]Univ Rennes, CNRS,
Geosciences Rennes, UMR 6118, F35000, France
*Corresponding author

1 Supplementary Methods

1.1 Magnetic field control

To control the orientation of the magnetotactic bacteria (MTB) within the microfluidic device, a magnetic field was generated via a customized set of Helmholtz coils, which was designed and fabricated to fit the objective and stage of the Nikon Ti-e microscope. The coils were fabricated from laser-cut acrylic and comprised $N \approx 100$ turns of 20 gauge copper wire. The coils had a 6 cm radius and 6 cm separation to ensure a uniform magnetic field. A computer controlled power supply (National Instruments VirtualBench) regulates the current, I, through the coils in the range $I \in [0, 500\ \text{mA}]$. The coils were capable of providing a magnetic field of strength, $B = kI \in [0, 1.75\ \text{mT}]$, with $k = 3.5 \pm 0.035$ mT/A, calibrated by a Tesla meter. The uncertainty here comes from the positioning of the Tesla probe: for example, in this case, corresponding to a 1 cm error in positioning the probe between the two Helmholtz coils.

1.2 Microfabrication

Corrugated microfluidic channels (height, $h = 110\ \mu$m) are comprised of semi-circular walls with alternating concavity (Fig. 1). The microchannels were fabricated from polydimethylsiloxane (PDMS) using standard soft lithography methods[1] and plasma bonded to standard, 1 mm thick glass slides.[2] Walls forming the constriction at the channel throat have a fixed radius of curvature, $R_t = 35\ \mu$m, and a fixed wall separation width, $w_t = 70\ \mu$m. The radius of curvature of the expansion region, R_p, which forms the pores, was varied, $R_p = [R_t, 2R_t, 3R_t]$, resulting in a pore width $w_p = w_t + 2R_t + 2R_p = [3w_t, 4w_t, 5w_t] = [210\ \mu\text{m}, 280\ \mu\text{m}, 350\ \mu\text{m}]$. For 3D porous media experiments (Fig. 4), a suspension of polydisperse, transparent agarose hydrogel beads (radius, 10-25 μm; fine grade, Agarose Beads Technologies) was packed into a microfluidic channel (height, 75 μm; width, 1000 μm). The channel exit had a 45° taper, which enabled the suspension to be jammed in the channel. After the packed bed was formed, the flow was switched to a suspension of magnetotactic bacteria under conditions described in the main text. Constant ambient flow maintained the arrested state of the bed for the duration of the experiment.

1.3 Microfluidics and pressure-driven flow calibration

To access the range of flow speeds relevant to the swimming magnetotactic bacteria within the microchannel throat, accurate control over the flow rate was required. A precision pressure controller (Elveflow OB-1) was used to drive the flow. The dynamic range of the applied pressure, P, was tuned by the addition of a serpentine resistor channel (height,

20 μm; width, 30 μm; total length, 10 cm) atop the corrugated test channel (Fig. S1). The resistor channels were plasma bonded to each test channel using standard methods.[2] We found that the resistor channels had a negligible effect on the bacterial image quality in the test channel.

For each experiment with magnetotactic bacteria, the physical control parameter for the cell dynamics is the maximal flow speed, U_{max}, in the throat of the corrugated test channel. Thus, the maximal flow speed was calibrated as a function of applied pressure, P, for each test channel. Fluorescent tracer particles (0.5 μm diameter) in cell media were flowed through each corrugated channel at five different applied pressures. The flow field was reconstructed from video images of the tracer particles using custom particle tracking velocimetry (PTV) routines (MATLAB), and the maximal flow speed was measured in each throat (Fig. S3a-c, blue circles). The resulting maximal flow speeds were fitted as a function of pressure with the expected linear relationship, $U_{max} = a_{max}P + b_{max}$, where the offset is due to hydrostatic pressure in the system (Fig. S3d-f, blue circles). The fitting coefficients are provided in Table S1. As a check, the same procedure was performed (Fig. S3d-f, red squares) for the minimal flow speed, U_{min}, along the channel centerline occurring in the pore (Fig. S3a-c, red squares). All experiments are performed in the low Reynolds number regime, ensuring linearity of the flow and pressure. In particular, the maximal Reynolds number of the bacterial experiments based on the channel height was $\mathrm{Re} = \rho U_{max} h/\eta \leq 0.05$, where ρ and η are the density and viscosity of water at room temperature, respectively. The ratio U_{max}/U_{min} was approximately constant to within $\pm 2\%$ for each geometry, consistent with the low Reynolds number flow conditions. For each experiment with cells, the corresponding U_{max} was computed from this calibration. Finite element simulations (COMSOL) corroborate our PTV measurements of the flow topology (Fig. S3g-i; see also below).

1.4 Langevin simulations in 1D porous media

Here, we illustrate the non-dimensionalization of the equations of motion for bacterial transport and discuss the key non-dimensional parameters that emerge, which are utilized throughout the main text. We then explain how the physical parameters characterizing magnetotactic motility are extracted from experimental data. We stress that there are no fitting parameters in the Langevin simulations, as all physical parameters are measured directly from experiments. Finally, we discuss the corresponding numerical simulation parameters.

1.4.1 Equations of motion for magnetotactic motility in flow

The Langevin equations of motion for magnetotactic bacteria in flow - originally introduced in the main text - are repeated here for convenience. This Langevin framework is well established for bacterial transport[3–5] and was recently adapted to magnetotactic bacteria.[6] The translational and rotational equations of motion in two dimensions are

$$\dot{x} = V_s \cos\theta + u(x,y), \tag{1}$$

$$\dot{y} = V_s \sin\theta + v(x,y), \tag{2}$$

$$\dot{\theta} = \frac{\omega(x,y)}{2} + \mu_r MB \sin(\theta) + \sqrt{2D_r}\xi(t). \tag{3}$$

Briefly, the orientation of the cells, θ, is measured relative to the upstream direction, x, and y is the cross-stream direction. The flow is described by the velocity field, $\mathbf{u} = (u,v)$, and the vorticity, $\omega = \nabla \times \mathbf{u}$. Bacterial motility is parameterized by the mean cell swimming speed, V_s, and rotational diffusivity, D_r, where the latter characterizes fluctuations in cell orientation due to both Brownian motion and noise in flagellar motility. The cells have a rotational hydrodynamic mobility, μ_r, and a magnetic moment, M, and they are exposed to a magnetic field, $\mathbf{B} = B\mathbf{e}_B$, oriented in the positive x-direction.

The equations of motion are non-dimensionalized in terms of the cell swimming speed, V_s, and the throat half-width of the channel, $R_{1D} = w_t/2$, as follows:

$$\frac{dX}{d\tau} = \cos\theta + \Phi\tilde{u}(X,Y), \tag{4}$$

$$\frac{dY}{d\tau} = \sin\theta + \Phi\tilde{v}(X,Y), \tag{5}$$

$$\frac{d\theta}{d\tau} = \Phi\frac{\tilde{\omega}(x,y)}{2} + \Delta\sin(\theta) + \sqrt{2\tilde{D}_r}\tilde{\xi}(\tau). \tag{6}$$

Here, the new dimensionless space and time variables are $X = x/R_{1D}$, $Y = y/R_{1D}$, and $\tau = tV_s/R_{1D}$. The linearity of the low Reynolds number flows considered here ensures that the velocity field topology does not change with the magnitude of the flow speed. The velocity field components and vorticity are thus scaled by the maximum value of the flow speed:

$$\tilde{u} = \frac{u}{U_{max}}, \tag{7}$$

$$\tilde{v} = \frac{v}{U_{max}}, \tag{8}$$

$$\tilde{\omega} = \omega \frac{R_{1D}}{U_{max}}. \tag{9}$$

The key non-dimensional numbers that emerge from the system are

$$\Phi = \frac{U_{max}}{V_s}, \tag{10}$$

$$\Delta = \frac{R_{1D} M B \mu_r}{V_s}, \tag{11}$$

$$\tilde{D}_r = D_r \frac{R_{1D}}{V_s}, \tag{12}$$

where Φ is a non-dimensional flow speed, Δ is a non-dimensional magnetic torque, and $\tilde{D}_r$ is a non-dimensional rotational diffusivity, or equivalently, an inverse rotational Péclet number. $\tilde{\xi}$ is a normalized white Gaussian noise.

1.4.2 Boundary conditions

Steric boundary conditions prevent cells from entering the solid surfaces in our simulations. We implement a Weeks-Chandler-Andersen repulsive potential:

$$\mathbf{F}_W(\mathbf{r}) = -\nabla U_r(\mathbf{r}) \tag{13}$$

$$U_r(d) = \begin{cases} 4\varepsilon\left[\left(\frac{a}{d}\right)^{1/6} - \left(\frac{a}{d}\right)^{1/12}\right] + \varepsilon, d < 2^{1/16}a \\ 0, d \geq 2^{1/16}a \end{cases} \tag{14}$$

where d is the distance to the closest solid surface, a is the bacteria radius. The hardness ε is set for its non-dimensional value $\tilde{\varepsilon} = \varepsilon/R_{1D}6\pi\eta a V_s = 2 \times 10^{-5}$. The radius of the bacteria is set to be $a = 0.5$ μm, amounting to a non-dimensional value $\tilde{a} = a/R_{1D} = 0.014$. This potential is zero away from the boundary and diverges fast as the cells gets closer to the surface. Cells are not oriented as the come close to the wall.

1.5 Experimental measurement of magnetotactic motility parameters

To ensure that the Langevin simulations are as physically representative of the experimental system as possible, all motility parameters of the swimming magnetotactic bacterial suspensions are measured directly, including D_r, V_s, and $\mu_r M$.

1.5.1 Rotational diffusivity

Micro-swimmers, including magnetotactic bacteria, experience a variety of noise in their swimming orientation, such as stochastic flagellar-actuated turning (e.g. run-and-tumble motility) and Brownian rotational diffusion. The resulting motility is often modeled as a continuous time random walk, where the noise is parameterized by an effective rotational diffusion, D_r. The rotational diffusion coefficient is measured by tracking a suspension of free-swimming magnetotactic bacteria (439 trajectories) in the absence of an applied flow and magnetic field. The autocorrelation function of bacterial orientation, $C_{ee}(t) = \langle \mathbf{e} \cdot \mathbf{e}(t) \rangle$, captures the persistence of the cell swimming direction, $\mathbf{e}$, which decays with time (Fig. S2a). The decay or persistence time, τ_p, for these cells was measured from an exponential fit to $C_{ee}(t)$ and found to be $\tau_p = 4$ s. The persistence time is directly related to the rotational diffusion coefficient as $D_r = 1/(2\tau_p) = 0.125$ s^{-1}.

1.5.2 Swimming speed

The mean swimming speed of the magnetotactic bacteria is also obtained by tracking a suspension of cells (> 1500 trajectories over 1 s long) in the absence of external flow, but with an applied magnetic field, comparable to the field strength used in the main text ($B = 0.875$ mT). The external magnetic field ensures that the main component of the swimming speed is co-planar with the imaging plane, and thus, there is no bias introduced by measuring a 2D projection of the 3D cell swimming velocity. The swimming speed distribution of the cells (Fig. S2b) had a mean value of $V_s = 135$ μm/s and a standard deviation of 25 μm/s. The magnetotactic bacteria are robust swimmers, and we verified that their mean swimming speed decreases by only 20% over the course of one hour. Such variations in swimming speed have minimal impact on the relatively brief 30 minute experiments presented in the main text.

1.5.3 Magnetic alignment

Several techniques were previously established to estimate the magnetic moment, M, of magnetotactic bacteria.[7] These methods typically involve the measurement of the orientation of motile or non-motile cells as an imposed magnetic field is varied. Because the magnetic momentum is not perfectly aligned, trajectories will take an helical shape,[8] but their direction, when averaged over a few pitches, will follow the magnetic field. However, the coccoid MC-1 bacteria used in this study are spherical and small in diameter ($d \approx 1$ μm), preventing direct optical measurement of the cell orientation. Furthermore, we also require the hydrodynamic rotational mobility, μ_r, of the cells to solve the Langevin equations of motion. The mobility may be estimated analytically by assuming a spherical cell, but $\mu_r \sim d^{-3}$ potentially leading to large errors, and this approach ignores the hydrodynamic contribution of the flagella. Recognizing that the rotational mobility and magnetic moment only appear together in the equations of motion, their product, $\mu_r M$, is easily measured from controlled cell tracking experiments. We measure the ensemble average alignment of the swimming direction, $\mathbf{e}$, with the magnetic field direction, $\mathbf{e}_B$, as $\langle \mathbf{e} \cdot \mathbf{e}_B \rangle$ for a range of magnetic field strengths, B. The result is predicted to obey the Langevin paramagnetic law:[7]

$$\langle \mathbf{e} \cdot \mathbf{e}_B \rangle = L(B/B_0) = \coth\left(\frac{B}{B_0}\right) - \frac{B_0}{B}, \tag{15}$$

where $B_0 = D_r/\mu_r M$. The magnetic alignment of the cells is hence measured by tracking an ensemble of bacteria under a range of magnetic field strengths and computing the mean relative alignment, $\langle \mathbf{e} \cdot \mathbf{e}_B \rangle$, which indeed follows the Langevin paramagnetic law (Fig. S2c). The result is fitted by the Langevin function to extract the characteristic magnetic field $B_0 = 45$ μT, which is relatively close to Earth's magnetic field of 56 μT. Because the rotational diffusivity of the cells is measured independently (see above), we can obtain an estimate for the product of the magnetic moment and rotational hydrodynamic mobility as $\mu_r M = D_r/B_0 = 2800$ T^{-1}s^{-1}.

1.6 Simulation parameters

To demonstrate this first observation of vortical localization of upstream swimming cells in porous media flows, we vary both the geometry and the flow strength, while holding the magnetic field fixed ($B = 1.75$ mT). The relevant non-dimensional magnetic alignment parameter is thus $\Delta = R_{1D} M B \mu_r / V_s = 1.25$, and the non-dimensional rotational diffusion coefficient is $\tilde{D}_r = D_r R_{1D}/V_s = 0.032$ for all simulations. The non-dimensional flow strength is varied in the range $\Phi = U_{max}/V_s \in [0, 10]$, and the geometries tested had pore to throat aspect ratios corresponding to experiments, including $w_p/w_t = [3, 4, 5]$, as well as straight channels.

Experimental particle tracking velocimentry (PTV) measurements of the channel flow fields (Fig. S3a-c) are used primarily to calibrate the maximal flow strength, U_{max}, as a function of applied pressure, P, (Fig. S3d-f) and to validate finite element simulations (COMSOL) of the flow fields (Fig. S3g-i). For Langevin simulations of the bacterial dynamics, we rely on the finite element simulations of the flow fields in each geometry. These two-dimensional, finite element simulations are a good approximation to the experimental flow fields, especially at the high-aspect ratio throat, and provide higher resolution of $u(x,y)$, $v(x,y)$, and $\omega(x,y)$. The finite element simulations are computed in the 2D channel domain using Stokes equation, $0 = -\nabla p + \eta \nabla^2 \mathbf{u}$, and continuity, $\nabla \cdot \mathbf{u} = 0$, with a no slip boundary condition on the corrugated walls for a single, arbitrary applied pressure gradient for each geometry. The finite element simulation domain consisted of 11 spatial corrugation periods with prescribed constant pressure conditions at the inlet and outlet, and it was meshed with over 70,000 vertices in each geometry. The velocity field from the most central corrugated pore is used as the periodic flow field for the Langevin simulations, and the vorticity, ω, is computed

directly from this flow field. Linearity enables the simulated flow field and vorticity field topologies to be scaled with the maximal centerline flow speed, U_{max}, for each Φ tested.

For the Langevin simulations, the non-dimensional time step was fixed at $\delta\tau = 0.005$. Time integration was performed by a fourth-order stochastic Runge-Kutta scheme, and the flow and vorticity fields were interpolated at each time step by a bilinear interpolation. Periodic boundary conditions were implemented in the stream-wise direction. For each condition, we simulated 600 cell trajectories for a total dimensionless simulation time, $\tau \geq 20{,}000$.

2 Supplementary Discussion

2.1 Influence of the magnetic field strength on the bacterial conductivity

A magnetic field of 1.7 mT was used throughout our experiments, which resides between the fields used in Magnetic Resonance Imaging ($\approx$ 1 T) and in environmental conditions ($\approx$ 56 μT), and is close to the 2 mT used in foreseen biomedical applications.[9] This relatively large field compared to the Earth Magnetic Field (EMF) ensured that cell alignment perpendicular to the image plane was negligible in our experiments. Based on the strong agreement between our experiments and Langevin simulations, we thus use the simulations to investigate the impact of the magnetic field strength on the transport of MTB in a corrugated channel corresponding to Figs. 1-2. The three cell transport regimes (upstream, trapping, and downstream) are clearly observable with significantly weaker magnetic fields (Fig. S6f-t) ranging from the experimental conditions (30 EMF) down to Earth's magnetic field (1 EMF). This reduction in the field strength marginally reduces the flow speed range of the plateau in cell flux for the trapping regime and the trapping strength as measured by the escape rate (Fig. S6a-e).

2.2 Influence of the magnetic field orientation on the bacterial conductivity

In our experiments, we used a magnetic field direction oriented anti-parallel to the flow. While this idealized system yields striking observations of dynamical cell trapping, in general, the flow direction may have an arbitrary orientation relative to the field, for example in environmental conditions or biomedical applications. We thus exploit Langevin simulations to determine the effect of the magnetic field orientation relative to the flow on the trapping phenomena demonstrated (Fig. S7) in the main text. In the corrugated channels, without flow, cells are directed by the magnetic field and guided by mechanical interactions with walls (Fig. S7f-j). When cells are directed into a concave wall of the corrugated channel, their random walk motility relative to the field strength is generally insufficient to enable escape from the wall. However, this idealized geometry was specifically designed to observe the vortical trapping effect illustrated in the main text (Fig. 1), which can be clearly observed for moderate flows (Fig. S7k-o). The bias in cell swimming direction creates an asymmetry in the vortical structure, which only appears on one side of the throat. By driving the cells on the side of the corrugated channels where the fluid velocity is also weaker, we observe that the plateau of the trapping regime is significantly extended (Fig. S7a-e), and larger flow rates are needed to wash away the cells and enter the downstream regime (Fig. S7p-t). In conclusion, the trapping regime is increased by the introduction of a relative angle between the magnetic field and flow direction, it is because of a cooperative effect between (*i*) the vortical trapping, which still occurs in these tilted configurations, (*ii*) steric trapping where cells are driven against the concave boundary of the channel (reducing the upstream regime), and (*iii*) their localization in a low flow speed region of the channel (reducing the downstream regime; Fig. S7a-e).

2.3 Langevin simulations in 2D porous media and the effect of disorder

2.3.1 Geometry, flow, and Langevin simulations

In this section, we use the numerical Langevin model detailed above to study in detail the the effects of disordered porous media in higher spatial dimension on cell transport for swimming magnetotactic bacteria directed upstream against a fluid flow.

The geometries considered here are based on an array of circular obstacles[5] (Fig. S8a). We consider both an ordered and a disordered arrangement, where the ordered geometry comprises a 10×10 square lattice of cylinders with porosity 0.5. The cylinders have radius, $R_{2D} = 105$ μm, which is three times the half-width of the throat for the 1D corrugated system, R_{1D}, with a center to center distance $a_{2D} = 8R_{1D} = 280$ μm. The wall-to-wall distance between two neighboring obstacles is hence equal to the throat width of the 1D corrugated medium, $2R_{1D} = 70$ μm.

The disordered medium comprises the same number of obstacles and same porosity as the ordered medium, but obstacles are randomly distributed (non-overlapping; Fig. S8b).

For the purposes of simulations, the disordered and ordered media are made globally periodic by tiling the obstacle geometry in a 3×3 array. Flow fields are computed numerically for each medium using Stokes flow assumptions in COMSOL as detailed above. The 3×3 array was placed in the middle of a rectangular channel with a length of 161.2 mm and width of 81.2 mm leaving an open space with a length of 38.95 mm both at the inlet and outlet of the channel. The maximum Reynolds number in the 2D porous geometries was Re ≤ 0.1, which suggests that the entrance length was L_{ent} = 0.05 LRe $\leq$ 0.35 μm for the highest flow speed examined, where L is taken as the wall-to-wall distance between two neighboring obstacles in the ordered lattice. The entrance length was thus sufficiently smaller than the length of the open space at the inlet as well as the length of a single tiling of the pillar arrangement (*i.e.* each one of the 3×3 array) ensuring a fully developed flow profile at the central portion of the 3×3 array. The simulations used a combination of triangular and quadrilateral mesh elements (2,605,814 elements for ordered and 4,111,650 elements for disordered geometry), which was greatly refined near the pillar surfaces (≈ 2 μm) to resolve the high velocity gradients. The upstream boundary condition was a constant unidirectional flow imposed at the channel inlet, and the downstream boundary condition at the outlet was a reference value for the pressure set to zero. No-slip boundary conditions were implemented on the pillar surfaces and slip boundary conditions on the sidewalls of the channel.

The central tile of the 3×3 flow field was used for Langevin simulations with periodic boundary conditions for both the fluid flow and simulated cell dynamics. For Langevin simulations, 2500 cells are initially placed homogeneously in the porous media in the disordered geometry, (400 cells in the ordered geometry). Their motion is then simulated via the Langevin equations above using the same Runge-Kutta scheme, same wall/obstacle boundary conditions, same time steps, and the same (non-dimensional) total simulation time $\tau = tV_S/R_{1D} = 256$ for the disordered geometry, and $\tau = tV_S/R_{1D} = 64$ in the ordered geometry (See 9). The sample scale conductivity at long times is defined from the ensemble speed average $\langle V(t_f)\rangle$ at the final time t_f as $\langle V(t_f)\rangle/V_S$.

2.3.2 Population dynamics reveal that heterogeneous flow in disordered media enhances trapping at the sample scale

Due to the geometrical uniformity of the flow field in the ordered media, all pores/throats uniformly exist in one of the three identified transport regimes (Fig. 4, Fig. S9a-b) corresponding to the 1D case (Fig. 2). However, the heterogeneity of the flow field in the disordered geometry enables the coexistence of the upstream, downstream, and trapping regimes (Fig. 4, Fig. S9c-d). To elucidate the role of disorder, we take a cell population dynamics approach. We associate each simulated cell with an instantaneous transport state – upstream, trapped, or downstream – depending on its velocity over $3R_{2D}/V_s$. Cells with a net displacement $\Delta X < -R_{2D}/2$ during this time are classified as in the downstream regime, while cells with a displacement $\Delta X > R_{2D}/2$ are classified as in the upstream regime. In between, cells are classified as being in the trapped regime, $-R_{2D}/2 \geq \Delta X \geq R_{2D}/2$. The evolution of these cell populations from an initially spatially uniform distribution is then studied during the simulations (Fig. S9, top row). We represented the initial and final spatial distributions of the cells and their states for three different dominant sample scale conductivity regimes for both geometries, *i.e.*, $\langle V\rangle/V_S(t_\infty) = 0.5$, 0, and -0.5 (Fig. S9, rows 2-4).

In their initially uniformly seeded positions, cells coexist in three corresponding populations over a wide range of flow speeds in the disordered geometry (Fig. S9c, top row), when compared to the ordered geometry (Fig. S9a, top row). In the ordered geometry, individual cells quickly settle into the regime associated with the sample scale due to the uniformity of the flow topology across pores, for example the trapping regime shown in Fig. S9a-b (row 3). In contrast, the state of the cell populations in the disordered geometry evolve more slowly: As cells circulate and sample the heterogeneous flow field (Fig. 4), they have a propensity to become trapped in specific pores, for example Fig. S9c-d (row 3). The proportion of trapped cells increases over time for all flow regimes investigated (Fig. S9c-d, rows 2-4). This feature emphasizes the fact that the residency time within the trapping regions becomes very long compared to any other time scales involved in cell transport, including upstream or downstream excursions. Cells circulate fast through these pores in the upstream and downstream states and conversely, reside for an extended time in trapping pores. Even though the trapping is a pore scale effect, the spatial correlations of the flow field (larger than the pore scale) translate into patches of pores in the same regime (Fig. S9c, third row, blue patch). Thus, while the nonlinear conductivity is a pore scale effect, the correlated heterogeneity of porous media flows results in spatial correlation of the bacterial conductivity.

3 Supplementary Tables

Supplementary Table 1: Fitting coefficients for the maximal, U_{max}, and minimal, U_{min}, flow speed versus pressure drop curves (Fig. S3d-f) for each corrugated microchannel pore geometry with an auxillary resistor channel.

Fit Coefficient	Pore Width, w_p		
	210 μm	280 μm	350 μm
1. a_{max} (μm/s/mbar)	5.7	5.92	5.6
2. a_{min} (μm/s/mbar)	4.24	3.17	2.33
3. b_{max} (μm/s)	7.9	27.1	36.5
4. b_{min} (μm/s)	12.33	16.96	12.2

4 Supplementary Figures

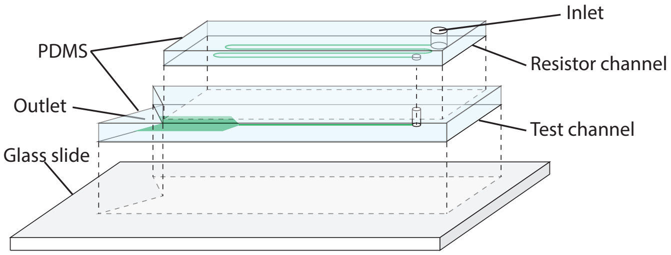

Supplementary Figure 1: Schematic of the corrugated microfluidic device and resistor channel. A serpentine resistor channel (height, 20 μm; width, 30 μm; total length, 10 cm) regulates the dynamic range of the applied pressure, and thus, controls the flow speed within the corrugated test channel. The resistor and corrugated test channels are both fabricated from PDMS. The outlet of the resistor channel is aligned with the inlet of the corrugated channel, and the two PDMS structures plasma bonded together. The resistor and corrugated test channels are also bonded to a standard glass microscope slide (thickness, 1 mm; not to scale).

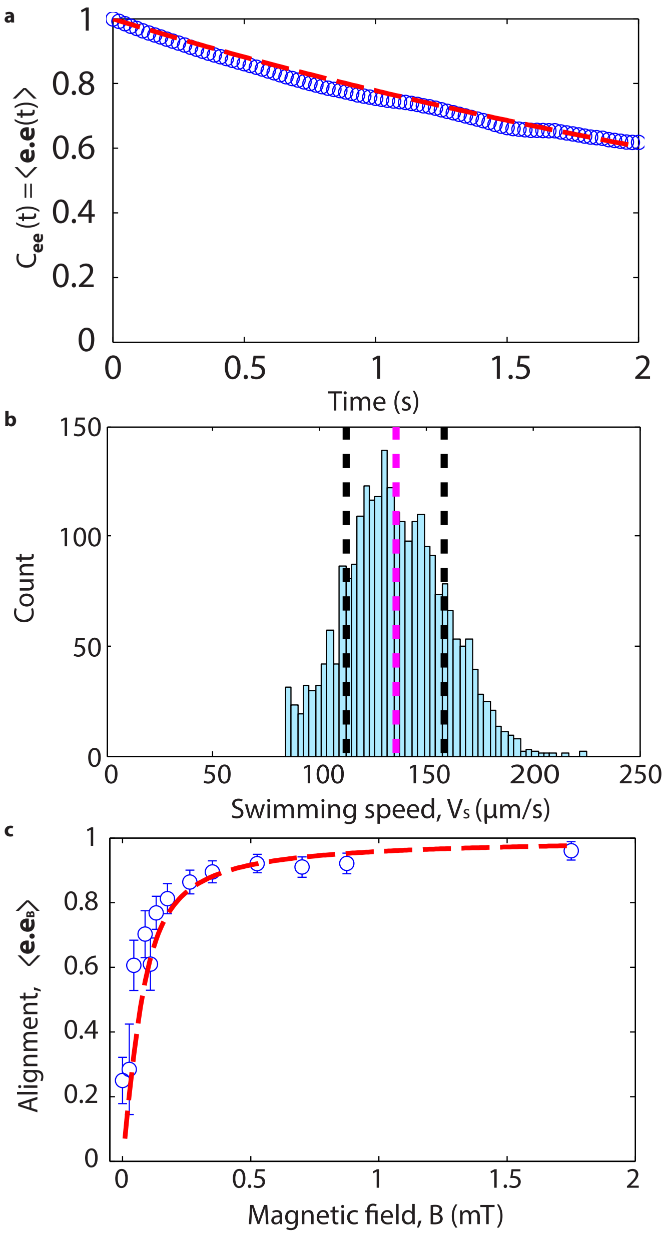

Supplementary Figure 2: Quantification of magnetotactic motility parameters for *Magnetococcus marinus* (MC-1). a, In the absence of an applied magnetic field and external flow, the cells undergo a persistent random walk. The effective rotational diffusivity, D_r, of the cells (due to both Brownian motion and noise in the flagellar kinematics) is measured from an exponential fit of the autocorrelation of their swimming direction, $\mathbf{e}(t)$, yielding a persistence time, $\tau_p = 1/(2.D_r) = 4$ s. **b**, The swimming speed is measured from the ensemble average of a population, $V_s = \langle V \rangle = 135 \pm 25$ μm/s (dashed magenta line) in the presence of the magnetic field comparable to experiments in the main text ($B = 0.875$ mT) and the variation is quantified by the standard deviation (dashed black lines). **c**, The degree of alignment of the bacterial orientation, $\mathbf{e}$, to the magnetic field direction, $\mathbf{e}_B$, is quantified by the ensemble average, $\langle \mathbf{e} \cdot \mathbf{e}_B \rangle$ (error bars correspond to the standard deviation). The mean alignment is well fitted with a Langevin function, $L(B/B_0) = \coth(B/B_0) - B_0/B$ (dashed red line). This established method[7] enables the extraction of the characteristic magnetic field for alignment, $B_0 = D_r/M\mu_r = 45$ μT. Each data point comprises the mean over 3472 cell trajectories on average, with a minimum sample size of 2391 trajectories.

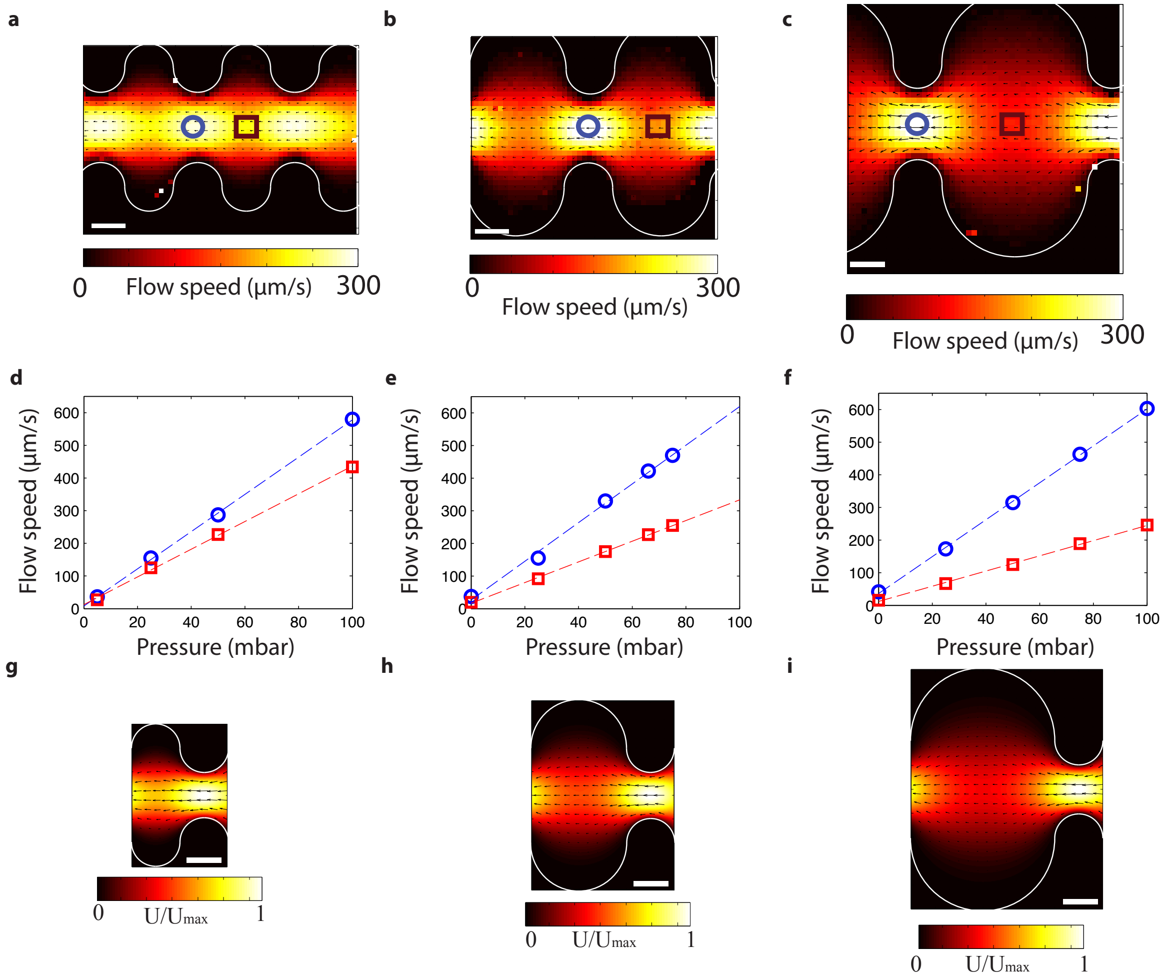

Supplementary Figure 3: Flow field measurement, calibration, and simulation. **a-c**, Particle tracking velocimetry (PTV) reconstruction of the flow field in the corrugated channels having pore widths, $w_p = [210\ \mu\text{m}, 280\ \mu\text{m}, 350\ \mu\text{m}]$, at respective pressure drops $P = [100\ \text{mbar}, 25\ \text{mbar}, 75\ \text{mbar}]$. The blue circles and red squares indicate locations of the maximum, U_{max}, and minimum, U_{min}, flow speeds along the channel centerline, occurring in the throats and pores, respectively. Wall locations are approximate. Scale bars, 35 μm. **d-f**, For each of the three corrugated microchannels, the flow strength was calibrated with the applied pressure for both the maximum and minimum centerline flow speeds. A linear fit to five experimental measurements, $U_{min,max} = a_{min,max}P + b_{min,max}$, provides the calibration coefficients, which are listed in Table S1. **g-i**, Finite element simulations (COMSOL) of the flow field for each of the three corrugated channels were performed in the low Reynolds number Stokes flow limit. The simulated flow fields corroborate PTV measurements and are utilized in subsequent Langevin simulations. Scale bars, 35 μm.

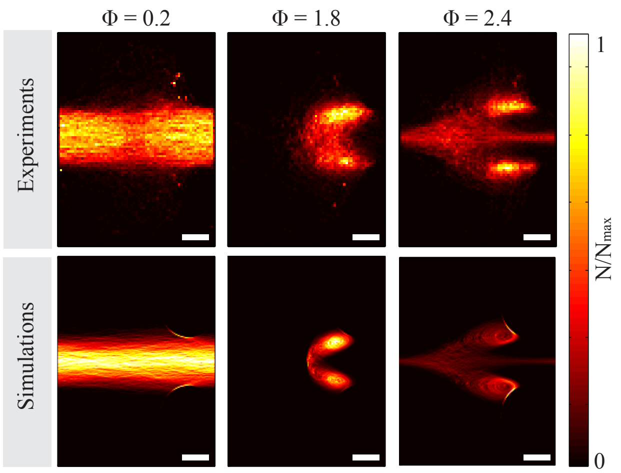

Supplementary Figure 4: Spatial distribution of upstream swimming magnetotactic cells in a corrugated microchannel flow. The relative cell count, N/N_{max}, in a corrugated microchannel (pore width, w_p = 280 μm) for the upstream ($\Phi = 0.2$), trapping ($\Phi = 1.8$), and downstream ($\Phi = 2.4$) flow regimes, for both experiments (top row) and simulations (bottom row). Results correspond to conditions and cell trajectories for experiments and simulations shown in Fig. 1d-i. Mean flow is from right to left, and magnetic field is from left to right. On average, each experimental flow condition presented comprises 3600 cell trajectories with a minimum of 2674 trajectories across the data set. Scale bars, 35 μm.

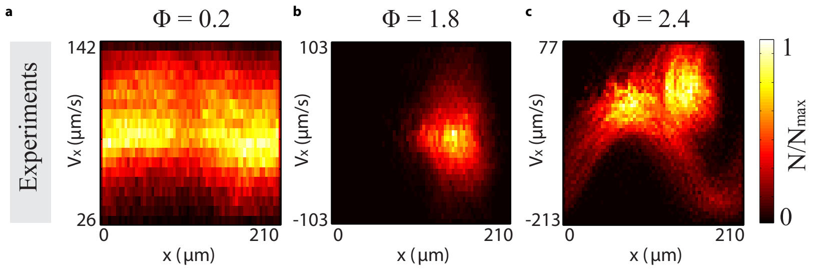

Supplementary Figure 5: Relative cell count in velocity-position phase space for upstream swimming magnetotactic cells in a corrugated microchannel flow. The experimentally measured relative cell count, N/N_{max}, in phase space for a corrugated microchannel (pore width, $w_p = 280$ μm) for the (**a**) upstream ($\Phi = 0.2$), (**b**) trapping ($\Phi = 1.8$), and (**c**) downstream ($\Phi = 2.4$) flow regimes. Results correspond to conditions for simulations in Fig. 3a-c. On average, each experimental flow condition presented comprises 3600 cell trajectories with a minimum of 2674 trajectories across the data set.

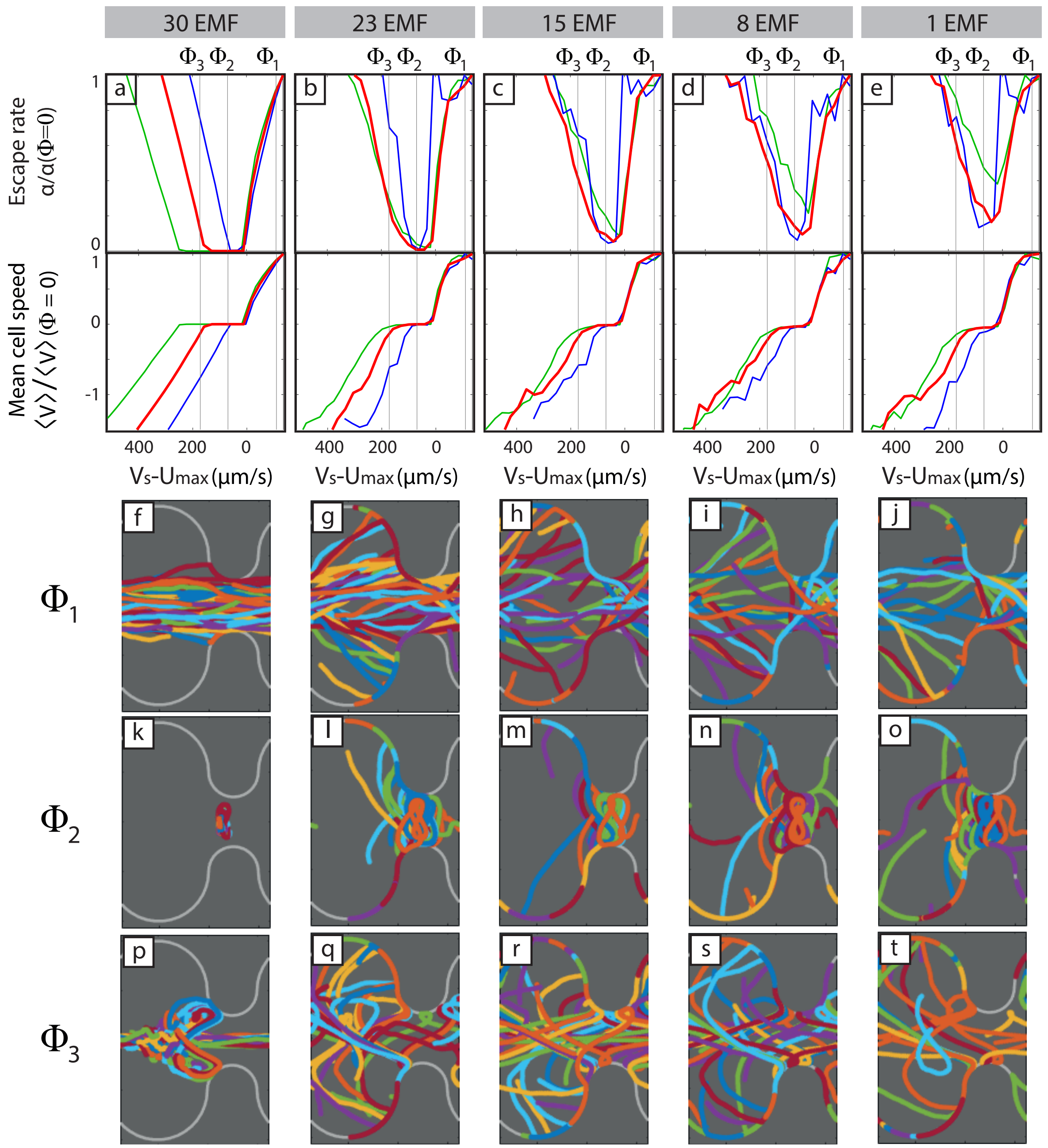

Supplementary Figure 6: Influence of magnetic field strength on pore scale transport. a-e, Langevin simulations reveal that a significant reduction in magnetic field strength from experimental conditions (30 EMF, 1.7 mT) to ambient field strength (1 EMF = 56 μT) results in only a modest reduction in the escape rate (top row) and the cell flux (mean cell speed) at the pore scale (bottom row). Green, red, and blue curves correspond to large, medium, and small corrugations (see main text). Vertical black lines mark the upstream (Φ_1), trapping (Φ_2), and downstream (Φ_3) regimes observed for 30 EMF in the medium corrugation (see also Fig. 2). **f-t**, For each of the three regimes defined in the main text, trajectories are represented for the medium corrugation corresponding to **a-e**.

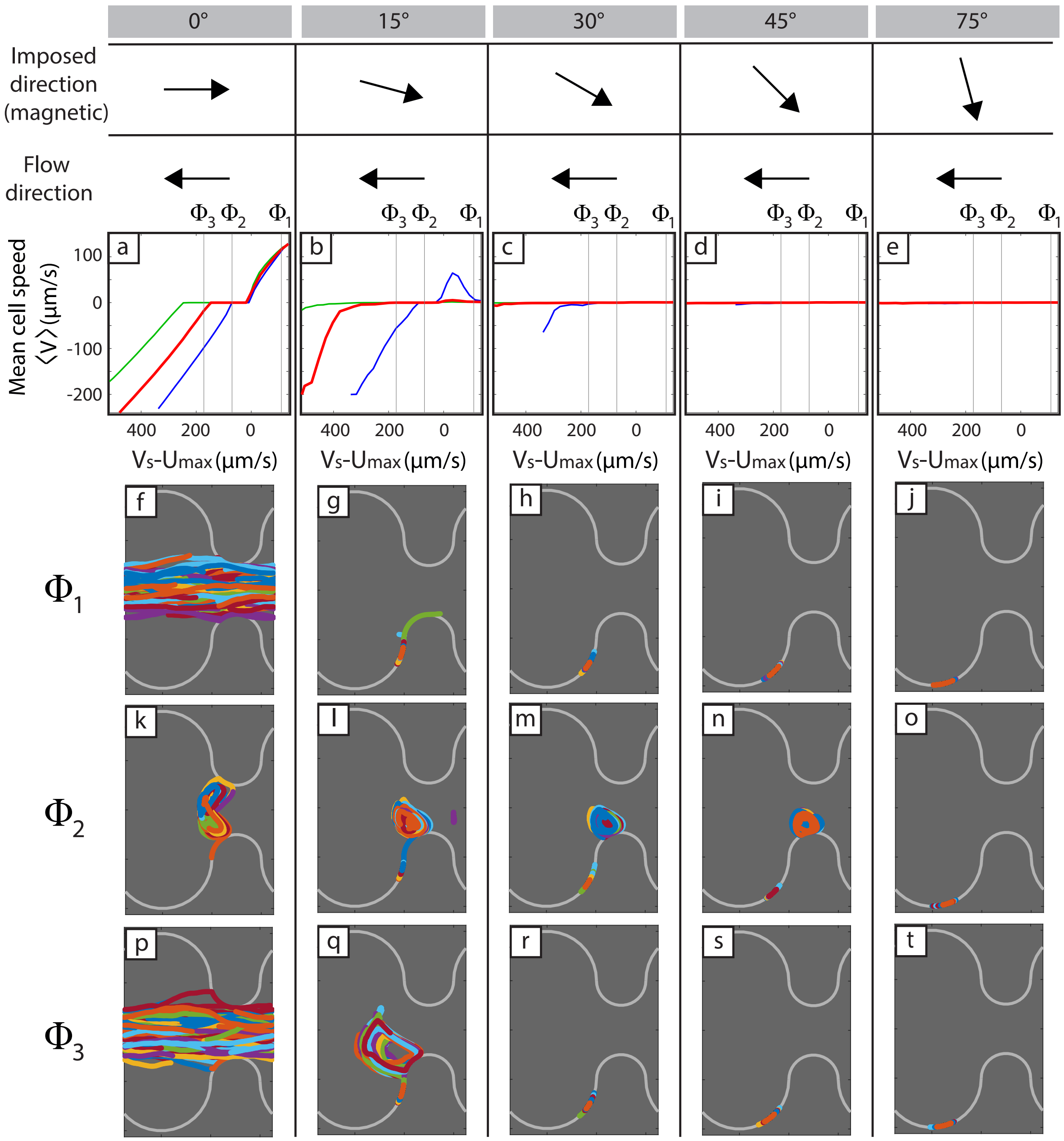

Supplementary Figure 7: Influence of magnetic field angle with the flow direction on pore scale transport. a-e, Langevin simulations reveal a systematic broadening of the trapping regime for the mean cell speed as the magnetic field angle increases relative to the flow direction for angles 0°, 15°, 30°, 45°, 75°. Green, red, and blue curves correspond to large, medium, and small corrugations (see main text). Vertical black lines mark the upstream (Φ_1), trapping (Φ_2), and downstream (Φ_3) regimes observed for 0° in the medium corrugation (see also Fig. 2). **f-t**, For each three regimes defined in the main text, trajectories are represented for the medium corrugation corresponding to **a-e**.

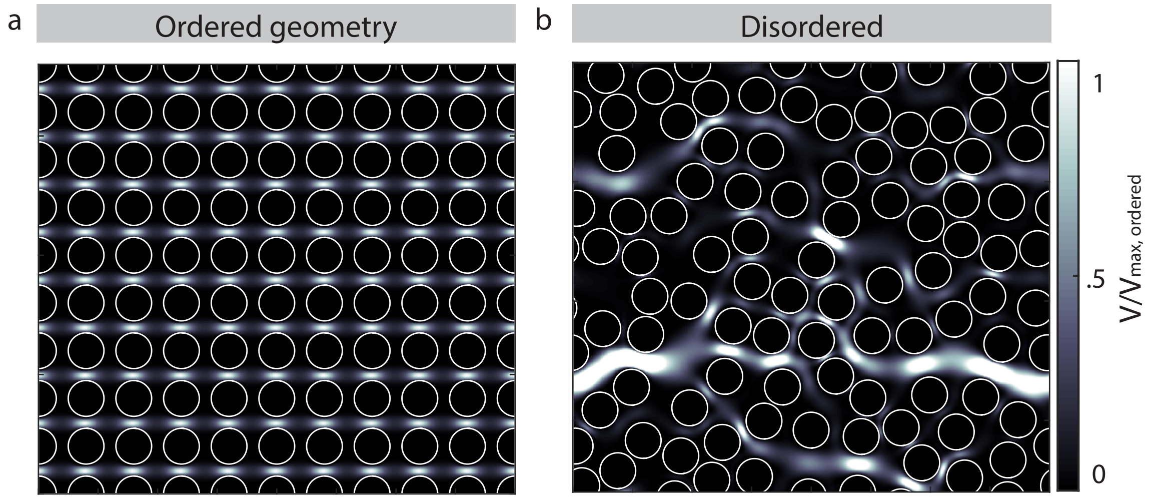

Supplementary Figure 8: Ordered and disordered flow fields. **a**, Flow field through the ordered geometry. **b**, Flow field through the disordered geometry. Color bar marks the flow speed normalized by the maximum flow speed in the ordered geometry. In the disordered geometry, the flow speed color map is saturated in this representation to show detail, since $V_{max,ordered} < V_{max,disordered}$.

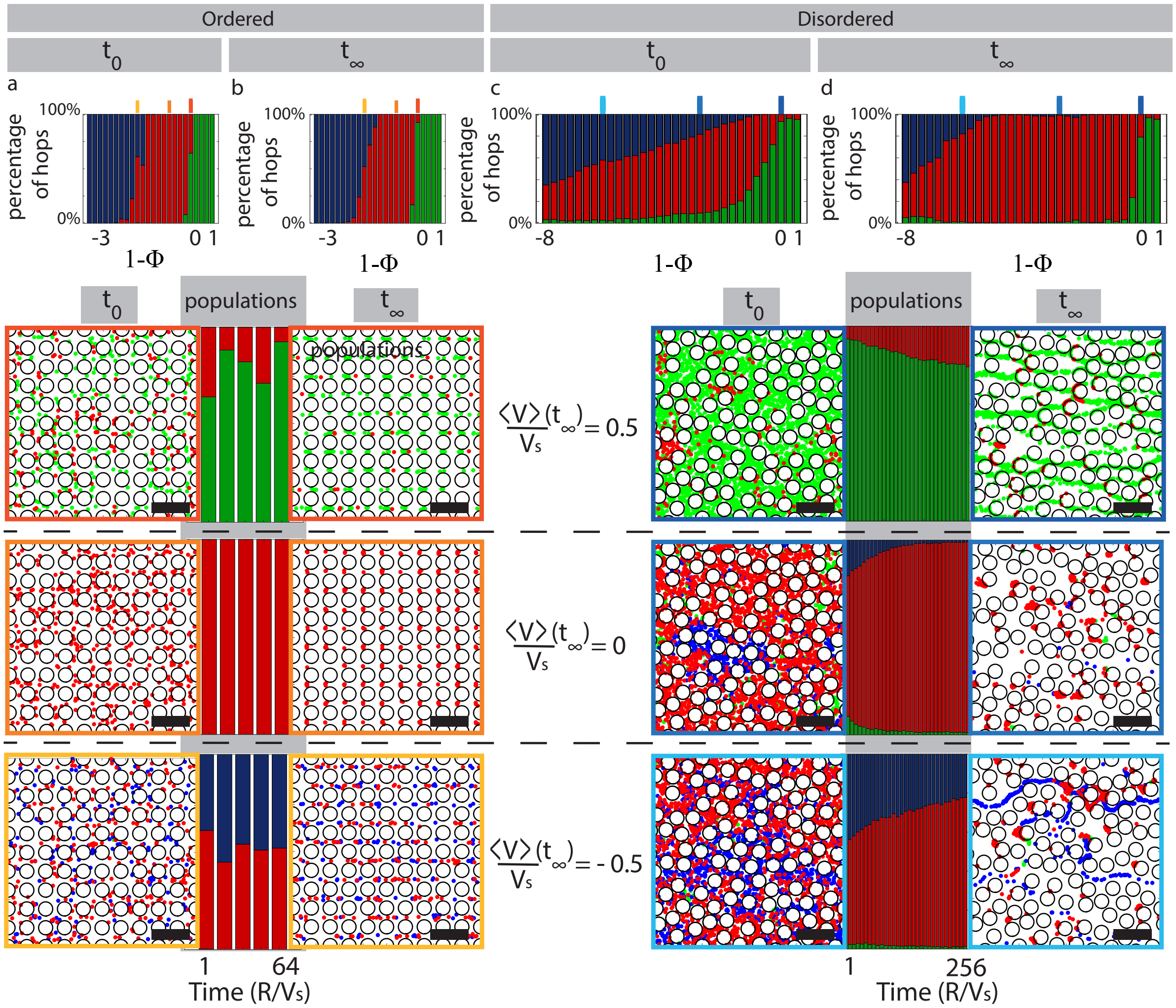

Supplementary Figure 9: Disorder promotes the coexistence of upstream, trapping, and downstream transport regimes **a-d**, (row 1) Populations of cells in the upstream (green), trapping (red), and downstream (blue) regimes. Initial (**a**, t_0) and final (**b**, t_∞) conditions for the ordered geometry and disordered geometry (**c-d**) are shown, respectively. Upper colored ticks indicate the average cell flow $\langle V \rangle / V_s$ rates value of 0.5, 0 and -0.5, colors corresponding to main text (Fig. 4-h). **a-d**, (row 2) Evolution of the cell population and cell locations in the upstream regime for the ordered geometry $\langle V \rangle (t_\infty)/V_s = 0.5$ (**a-b**) and for the disordered geometry (**c-d**). **a-d**, (row 3) Same as row 2 for the trapping regime $\langle V \rangle / V_s = 0$ as defined by the ordered geometry. **a-d**, (row 4) Same as rows 2-3 for the downstream regime $\langle V \rangle / V_s = -0.5$ as defined by the ordered geometry.

Supplementary References

[1] Xia, Y. & Whitesides, G. M. Soft lithography. *Annual Review of Materials Science* **28**, 153–184 (1998).

[2] Haubert, K., Drier, T. & Beebe, D. Pdms bonding by means of a portable, low-cost corona system. *Lab on a Chip* **6**, 1548–1549 (2006).

[3] Zöttl, A. & Stark, H. Nonlinear dynamics of a microswimmer in poiseuille flow. *Physical Review Letters* **108**, 218104 (2012).

[4] Rusconi, R., Guasto, J. S. & Stocker, R. Bacterial transport suppressed by fluid shear. *Nature Physics* **10**, 212–217 (2014).

[5] Dehkharghani, A., Waisbord, N., Dunkel, J. & Guasto, J. S. Bacterial scattering in microfluidic crystal flows reveals giant active taylor–aris dispersion. *Proceedings of the National Academy of Sciences* **116**, 11119–11124 (2019).

[6] Waisbord, N., Lefèvre, C. T., Bocquet, L., Ybert, C. & Cottin-Bizonne, C. Destabilization of a flow focused suspension of magnetotactic bacteria. *Physical Review Fluids* **1**, 053203 (2016).

[7] Nadkarni, R., Barkley, S. & Fradin, C. A comparison of methods to measure the magnetic moment of magnetotactic bacteria through analysis of their trajectories in external magnetic fields. *PloS One* **8**, e82064 (2013).

[8] Pan, Y. *et al.* Reduced efficiency of magnetotaxis in magnetotactic coccoid bacteria in higher than geomagnetic fields. *Biophysical Journal* **97**, 986–991 (2009).

[9] Martel, S., Mohammadi, M., Felfoul, O., Lu, Z. & Pouponneau, P. Flagellated magnetotactic bacteria as controlled mri-trackable propulsion and steering systems for medical nanorobots operating in the human microvasculature. *The International journal of robotics research* **28**, 571–582 (2009).
