# Supplementary material for: Fluidic bacterial diodes rectify magnetotactic cell motility in porous environments
Source: Nat Commun. 2021 Oct 12;12:5949. doi: 10.1038/s41467-021-26235-6 (PMC8511139; doi:10.1038/s41467-021-26235-6)
Supplement: Supplementary file 7 — Reporting Summary [file 41467_2021_26235_MOESM7_ESM.pdf]

nature portfolio

Corresponding author(s): Jeffrey S. Guasto

Last updated by author(s): Sep 15, 2021

Reporting Summary

Nature Portfolio wishes to improve the reproducibility of the work that we publish. This form provides structure for consistency and transparency in reporting. For further information on Nature Portfolio policies, see our Editorial Policies and the Editorial Policy Checklist.

Statistics

For all statistical analyses, confirm that the following items are present in the figure legend, table legend, main text, or Methods section.

n/a	Confirmed	
☐	☒	The exact sample size (n) for each experimental group/condition, given as a discrete number and unit of measurement
☐	☒	A statement on whether measurements were taken from distinct samples or whether the same sample was measured repeatedly
☒	☐	The statistical test(s) used AND whether they are one- or two-sided<br>*Only common tests should be described solely by name; describe more complex techniques in the Methods section.*
☒	☐	A description of all covariates tested
☒	☐	A description of any assumptions or corrections, such as tests of normality and adjustment for multiple comparisons
☐	☒	A full description of the statistical parameters including central tendency (e.g. means) or other basic estimates (e.g. regression coefficient) AND variation (e.g. standard deviation) or associated estimates of uncertainty (e.g. confidence intervals)
☒	☐	For null hypothesis testing, the test statistic (e.g. F, t, r) with confidence intervals, effect sizes, degrees of freedom and P value noted<br>*Give P values as exact values whenever suitable.*
☒	☐	For Bayesian analysis, information on the choice of priors and Markov chain Monte Carlo settings
☒	☐	For hierarchical and complex designs, identification of the appropriate level for tests and full reporting of outcomes
☒	☐	Estimates of effect sizes (e.g. Cohen's d, Pearson's r), indicating how they were calculated

Our web collection on statistics for biologists contains articles on many of the points above.

Software and code

Policy information about availability of computer code

Data collection	Data in the form of digital image sequences (videos) were acquired using Nikon NIS-Elements.
Data analysis	The data were analyzed using customized MATLAB codes.

For manuscripts utilizing custom algorithms or software that are central to the research but not yet described in published literature, software must be made available to editors and reviewers. We strongly encourage code deposition in a community repository (e.g. GitHub). See the Nature Portfolio guidelines for submitting code & software for further information.

Data

Policy information about availability of data

All manuscripts must include a data availability statement. This statement should provide the following information, where applicable:

- Accession codes, unique identifiers, or web links for publicly available datasets
- A description of any restrictions on data availability
- For clinical datasets or third party data, please ensure that the statement adheres to our policy

The figure source data files generated in this study have been deposited in the Harvard Dataverse database https://doi.org/10.7910/DVN/YR7CPE. The raw experimental data (cell trajectories in time) and simulation data are available from the corresponding authors upon reasonable request.

Field-specific reporting

Please select the one below that is the best fit for your research. If you are not sure, read the appropriate sections before making your selection.

☐ Life sciences ☐ Behavioural & social sciences ☒ Ecological, evolutionary & environmental sciences

For a reference copy of the document with all sections, see nature.com/documents/nr-reporting-summary-flat.pdf

Ecological, evolutionary & environmental sciences study design

All studies must disclose on these points even when the disclosure is negative.

Study description	We performed a laboratory study of the directed transport of magnetotactic bacteria in idealized (microfluidic) and realistic (packed bed) porous media, when the cells are directed to swim in the upstream direction of the flow via an applied magnetic field.
Research sample	A cultured laboratory strain of magnetotactic bacteria (Magnetococcus marinus, MC-1) is transfered to a microfluidic device for assays.
Sampling strategy	Separate cultures were prepared for each microchannel geometry and packed bed on different days. Each analyzed movie of several hundred to several thousand frames yielded thousands of cell positions comprising several hundred cell trajectories. These large sample sizes are standard.
Data collection	For a given channel geometry, applied flow speeds were sampled in random order and movies of cell motility were captured by co-author Nicolas Waisbord using commercially available Nikon NIS-Elements microscopy software.
Timing and spatial scale	For a given microfluidic channel geometry, all data was collected on the same day with an individual cell culture prepared on the day of the experiment. Imposed flow speeds were varied randomly all data were captured within 1 hour of transferring the cells to the microfluidic device to ensure cell motility was preserved. Furthermore, the order of imposed flow speeds were randomized to ensure the data were insensitive to any potential variations in cell motility in time.
Data exclusions	No data were excluded from analyses.
Reproducibility	All attempts to reproduce the experiment were successful as exemplified by the consistency of the observed effect in the various microfluidic channel geometries captured on different days with different cultures. The observed effect was also observed qualitatively and repeatedly during testing and development of the microfluidic channel.
Randomization	Samples were allocated randomly and measured on different days. Within an experiment, cells were sampled from several different, but identical pores in the microfluidic device (e.g. see Figure 2).
Blinding	Blinding was not relevant to our study, as we were measuring microorganism motility using automated tracking codes.

Did the study involve field work? ☐ Yes ☒ No

Reporting for specific materials, systems and methods

We require information from authors about some types of materials, experimental systems and methods used in many studies. Here, indicate whether each material, system or method listed is relevant to your study. If you are not sure if a list item applies to your research, read the appropriate section before selecting a response.

Materials & experimental systems

n/a	Involved in the study	
☒	☐	Antibodies
☒	☐	Eukaryotic cell lines
☒	☐	Palaeontology and archaeology
☒	☐	Animals and other organisms
☒	☐	Human research participants
☒	☐	Clinical data
☒	☐	Dual use research of concern

Methods

n/a	Involved in the study	
☒	☐	ChIP-seq
☒	☐	Flow cytometry
☒	☐	MRI-based neuroimaging
